# The new phthalic acid-based deep eutectic solvent as a versatile catalyst for the synthesis of pyrimido[4,5-*d*]pyrimidines and pyrano[3,2-*c*]chromenes

**DOI:** 10.1186/s13065-024-01227-x

**Published:** 2024-06-27

**Authors:** Arezo Monem, Davood Habibi, Abdolhamid Alizadeh, Hadis Goudarzi

**Affiliations:** 1 Department of Organic Chemistry and Petroleum Chemistry, Faculty of Chemistry, Bu-Ali Sina University, Hamedan, 6517838683 Iran; 2 Department of Organic Chemistry, Faculty of Chemistry, Alzahra University, Tehran, 1993893973 Iran

**Keywords:** Methyltriphenylphosphonium bromide, Phthalic acid, DES, Pyrimidopyrimidines, Pyrano-chromenes

## Abstract

A new DES (MTPPBr-PHTH-DES) was prepared from a mixture of methyltriphenyl-phosphonium bromide (MTPPBr) and phthalic acid (PHTH). The eutectic point phase diagram showed that a one-to-one molar ratio of MTPPBr to PHTH is the optimal molar ratio for the synthesis of new DES. Then, it was characterized with various techniques such as FT-IR, TGA/DTA, densitometer, eutectic point, and NMR and used as a novel acid catalyst in the synthesis of pyrimido[4,5-*d*]pyrimidines and pyrano[3,2-*c*]chromes in solvent-free condition. Short reaction time, low temperature, high efficiency, green condition, and easy recycling and separation of the DES catalyst are among the most important features of the presented method.

**Supplementary Information:**

The online version contains supplementary material available at 10.1186/s13065-024-01227-x.

## Introduction

The importance of environmental protection has recently been seriously emphasized, and green chemistry has followed a smooth path to achieving these goals. Targeted use of solvents and catalysts is a fundamental solution to pursue environmental goals [1, 2]. Green environment solvents must meet various criteria such as availability, non-toxicity, recyclability, thermal stability, renewable ability, non-flammability, low vapor pressure, economy, and biodegradability [3, 4].

DESs have been developed in line with the goals of green chemistry as a suitable alternative to ionic liquids (ILs) and have found many applications in various research fields [5]. DESs are often cheap and safe and usually consist of mixing two or three ionic compounds where each component has a high melting point, but when they are combined, the melting point of the prepared DES is lower than either component [6–8]. DESs have countless advantages over conventional solvents, as they not only comply with the principles of green chemistry but also act as catalysts, depending on their properties and they do not need to be separated and purified. The use of DESs as acid catalysts has several advantages, including nontoxicity, catalytic efficiency comparable to or better than that of the acid itself, and the possibility of recovery and reuse without significant loss of activity [9–11].

A multicomponent reaction (MCR) is a reaction in which three or more reagents are added simultaneously to a reaction flask and mixed in a one-pot process. These reactions have several advantages over conventional synthesis protocols, including fewer steps, and no need to separate reaction intermediates, resulting in fewer purification steps. Since most of the carbon atoms are present in the final product, MCR can be considered as a good atom-economical process [12–14].

Since pyrimidine moieties are present in the structure of many natural compounds, they have been studied for more than a century for their chemical and biological importance, including anti-oxidant, anti-inflammatory, immunomodulatory, anti-bacterial, anti-viral, anti-hypertensive, anti-cancer, anti-thyroid, anti-parasitic, anti-malarial, anti-HIV, anti-viral, antifungal, anti-Leishmania, anti-HCV, anti-tumor, and urease inhibitory activities [15–26].

**Scheme 1:**
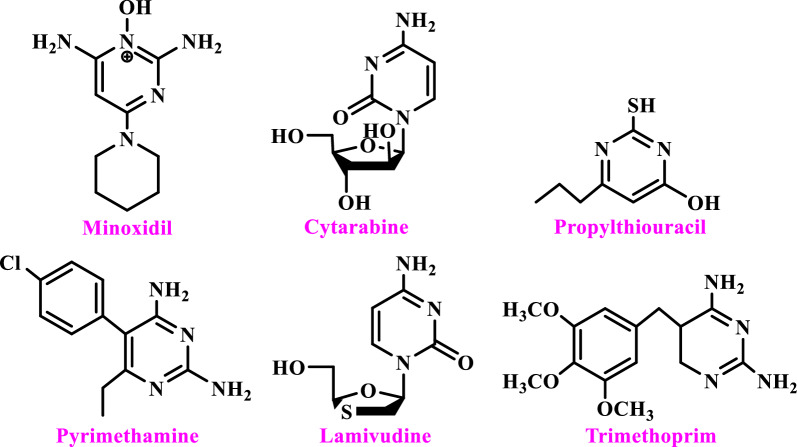
Structure of pyrimidine containing drugs

Below are some compounds that have a pyrimidine ring in their structure and are used in medicine. For example, Minoxidil acts directly on the smooth muscles of the vascular wall and reduces peripheral vascular resistance and blood pressure [27]. Cytarabine is easily converted into nucleotides inside the cells, inhibits DNA synthesis, and has a strong effect of suppressing the immune system and anticancer [22]. Propylthiouracil inhibits the synthesis of thyroid hormones and has antithyroid activity [23]. Primethamine has anti-infective and anti-malarial activity [24]. Lamivudine is an antiseptic drug and is used for HIV infections [25]. Trimethoprim is an antibiotic with a wide application range, which is used in the treatment of infections, especially urinary infections [25]. (Scheme 1).

Barbituric acids (BAs) are an important class of pyrimidines that have many medicinal uses and are used as hypnotics, sedatives, anti-convulsant, anesthetics, and anti-fungal. The most commonly used barbiturate drugs include butalbital, phenobarbital, barbital, and thialbarbital, and multicomponent reactions are the good method for the synthesis of heterocyclic compounds. High efficiency, short reaction time, energy saving, and simple operation are the advantages of multicomponent reactions [28–31].

**Scheme 2:**
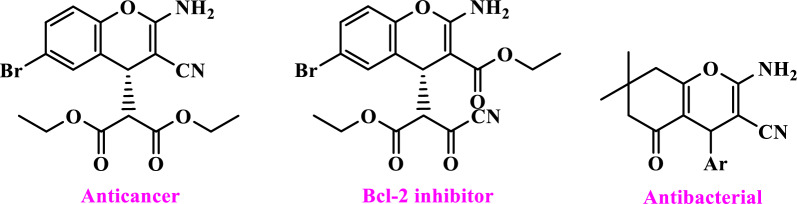
Structure of drugs containing chromenes

Chromenes are an important group of heterocyclic compounds that are the result of the fusion of a benzene ring with a pyran ring. As an important class of compounds, they are widely present in plants, including edible vegetables and fruits, and as drugs have significant effects, including anticancer, anti-HIV, antiviral, anticoagulant, anti-anaphylaxis, spasmolytic, and diuretic [32–37] (Scheme 2).

**Scheme 3:**
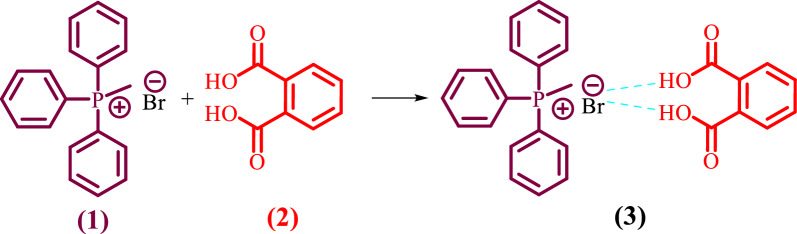
Synthesis of MTPPBr-PHTH-DES

Continuing our research on the preparation of novel catalytic systems, we would like to report here the preparation and characterization of the novel DES **(3)** by mixing one mole of MTPPBr **(1)** and one mole of PHTH **(2)** (Scheme 3).

**Scheme 4:**
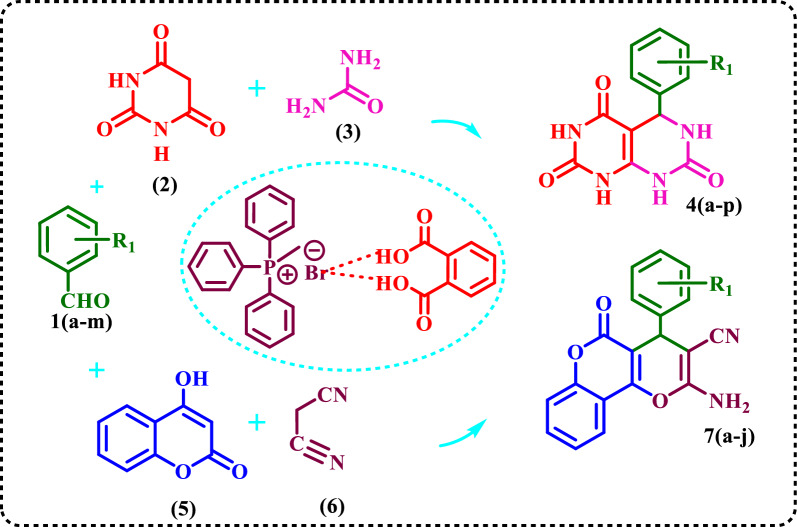
Synthesis of **4(a**–**p)** and** 7(a**–**j)** by DES

Then, it was used as a novel DES catalyst in the synthesis of two sets of the following compounds at 70 °C in solvent‐free condition:

- Pyrimido[4,5-*d*]pyrimidines **4(a**–**p)** from the reaction of aldehydes **(1)**, BA **(2)**, urea **(3)**, and
- Pyrano[3,2-*c*]chromenes **7(a**–**j)** from the reaction of 4-hydroxycoumarin **(5)**, malononitrile **(6)**, and aldehydes **(1)** (Scheme 4).

## Results and discussion

### Characterization of MTPPBr-PHTH-DES

The new DES was characterized by FT-IR, ^1^H NMR TGA-DTA, densitometer, and eutectic points.

### Characterization by FT-IR

**Fig. 1:**
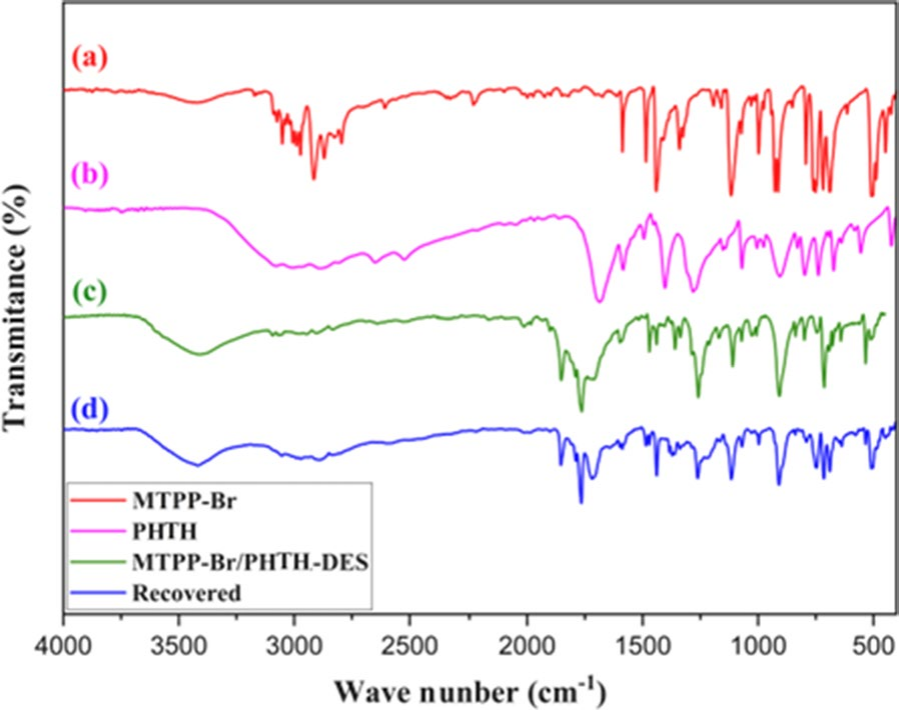
The FT-IR spectra of (**a**–**d**)

Figure 1 shows the IR spectra of MTPP-Br (a), PHTH (b), the fresh DES (c), and the recovered DES (d). In spectrum (a), the peaks at about 2900–3100 cm^−1^ are related to the aromatic and aliphatic hydrogens, and the peaks at about 750 and 1480 cm^−1^ are related to the C-P bonds, respectively. In spectrum (b), the peak in1700 cm^−1^ is related to the C=O group, and the broad peak in the region of 2400–3100 cm^−1^ is related to the OH of COOH. In spectrum (c), the indicated peaks can be seen in both (a) and (b) spectra, which confirm the structure of the DES catalyst.

To confirm the structure of the recovered DES (d), the corresponding IR spectrum was obtained which shows that there is no significant difference between the fresh (c) and the recovered IR spectra.

### Characterization by ^1^H NMR

#### The ^1^H NMR spectrum of MTPPBr

**Fig. 2:**
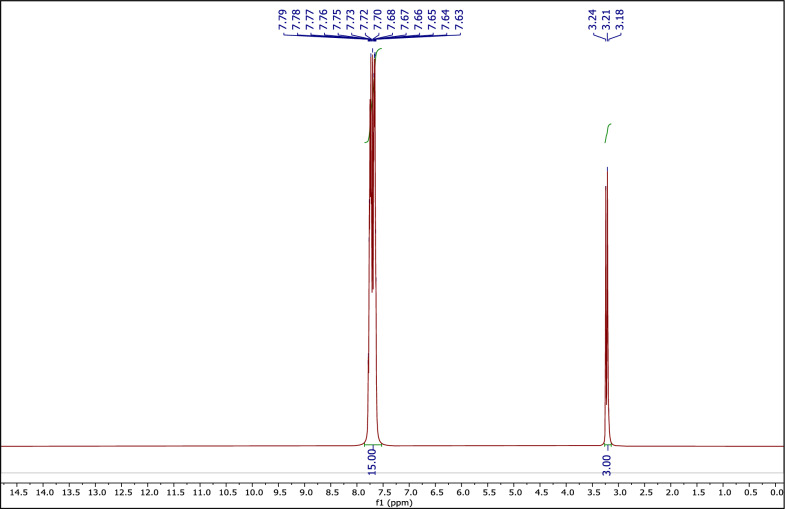
The ^1^H NMR of MTPPBr

Figure 2 shows the ^1^H NMR spectrum of MTPPBr. Peaks at 3.18–3.24 (d, 3H), and 7.79–7.63 (m, 15H) ppm are related to the CH_3_ hydrogens, and the three phenyl ring hydrogens of MTPPBr, respectively.

#### The ^1^H NMR spectrum of PHTH

**Fig. 3:**
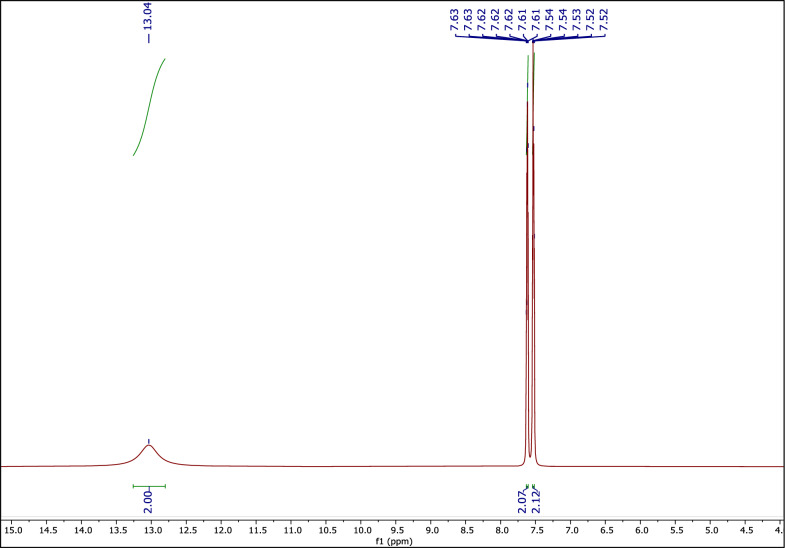
The ^1^H NMR of PHTH

Figure 3 shows the ^1^H NMR spectrum of PHTH. The peak at 13.04 (s, 1H) belongs to a hydrogen of the –COOH group. The peaks at 7.52–7.54 (d, 2H) and 7.61–7.63 (d, 2H) ppm are related to the four hydrogens of a phenyl ring.

#### The ^1^H NMR spectrum of MTPPBr-PHTH-DES

**Fig. 4:**
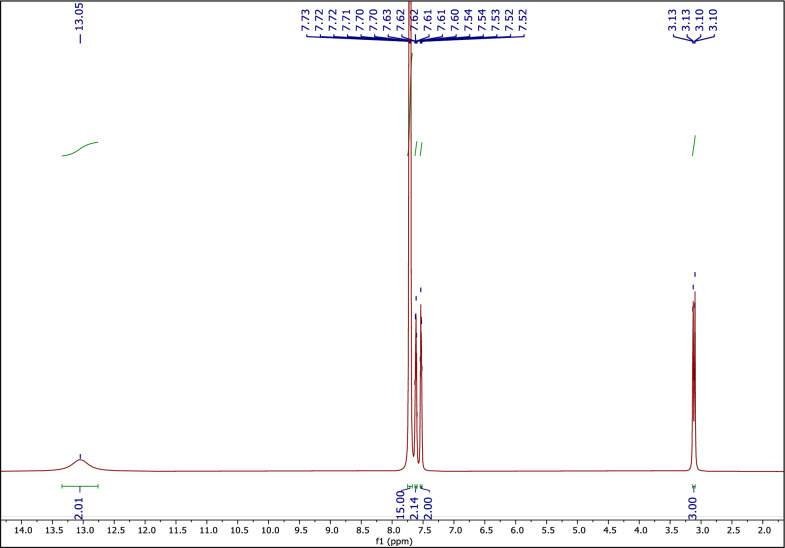
The ^1^H NMR of DES

Figure 4 shows the ^1^H NMR spectrum of MTPPBr-PHTH-DES. Peaks at 3.10–3.13 (d, 3H), 7.71 (dt, *J* = 10.0, 3.7 Hz, 15H), 7.61 (dq, *J* = 7.9, 4.1 Hz, 2H), 7.53 (dt, *J* = 5.9, 3.6 Hz, 2H) and 13.05 (s, 2H) ppm are related to the CH_3_ hydrogens, three phenyl ring hydrogens, the phenyl ring of PHTH, and the acid groups, respectively. When DES is formed, the signal intensity of hydrogens weakens and shifts towards the low field. These observations indicate the presence of the new hydrogen bond interactions between MTPPBr and PHTH [38], confirming the structure of the newly formed DES.

### Characterization by TGA-DTA

**Fig. 5:**
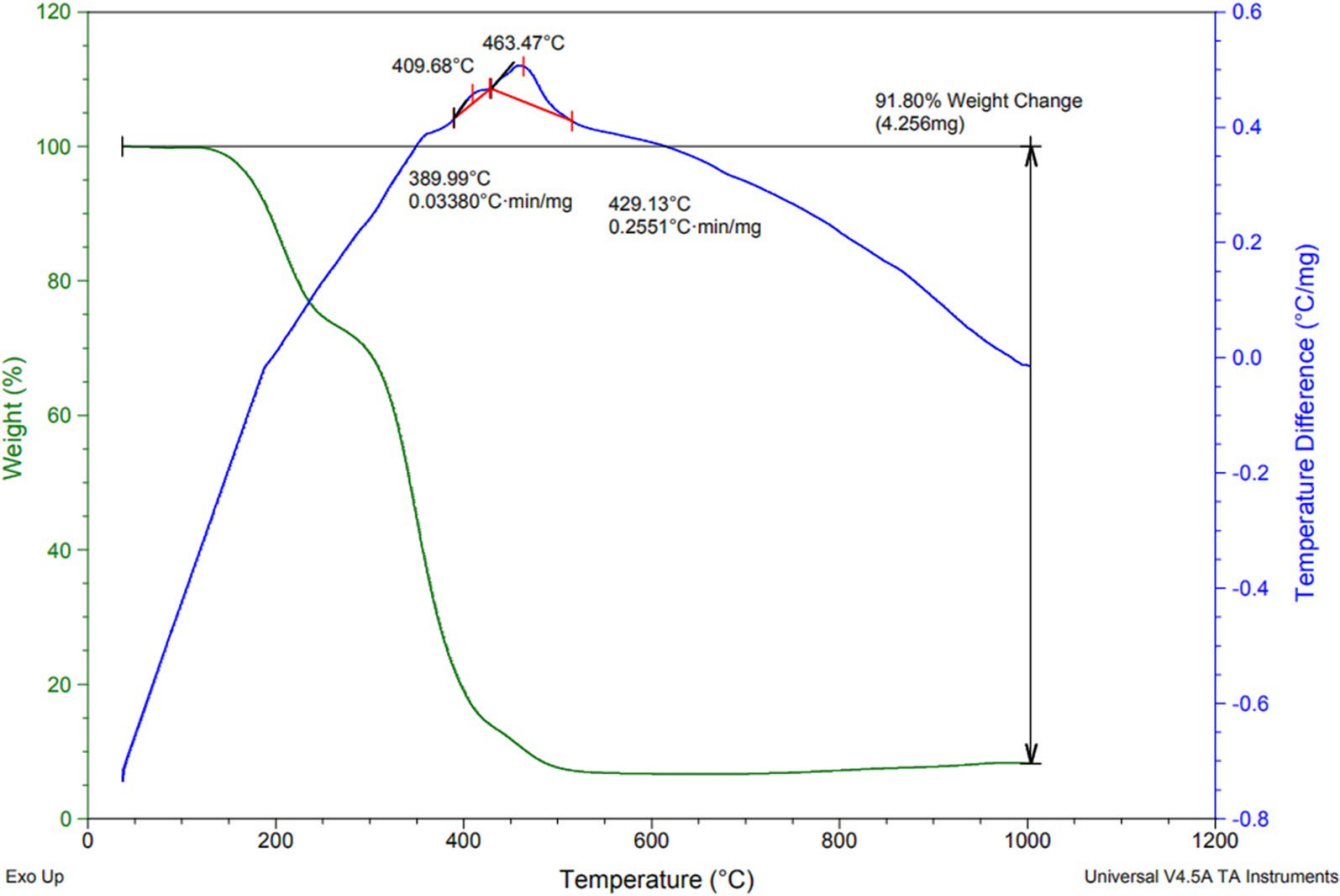
The TGA-DTA pattern of DES

To investigate the stability and thermal behavior of the new DES, the TGA-DTA analysis was performed which shows three breaks (Fig. 5). The first failure in the area below 200 °C is related to the absorbed vapors during preparation of the catalyst. The second break in the area below 400 °C is probably related to the breaking of hydrogen bonds in the DES structure and removal of the acidic group, and the break at 600 °C is related to the decomposition of MTPPBr.

### Characterization by densitometer

DESs usually have a density of 1.0 to 1.35 g/cm^3^, so a certain weight of DES was mixed with a certain volume of water, and its density was calculated using the relevant formula, which is about 1.33013 g/ml [39].

### Characterization by eutectic points

**Fig. 6:**
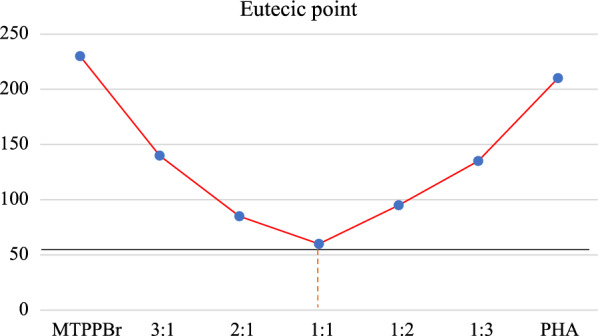
The Eutectic points phase diagram pattern of DES

To check the best ratio of MTPPBr to PHTH, the eutectic point experiment was performed, and different ratios of MTPPBr to PTHT were prepared. The eutectic point phase diagram (Fig. 6) showed that the best ratio for the novel DES formation is one mole of MTPPBr to one mole of PHTH.

The melting points of MTPPBr and PHTH are 230 and 210 °C, respectively, but when a novel DES was prepared, its melting point decreased to 60 °C.

### Optimization of the reaction conditions for the synthesis of 4h

**Scheme 5:**
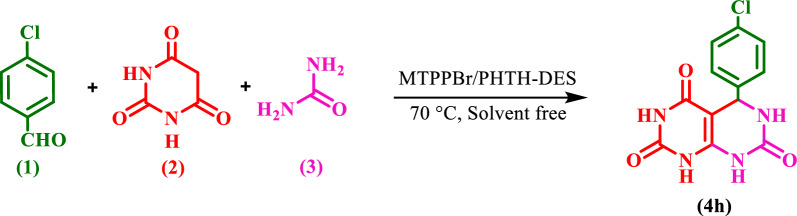
Synthesis of **4h** by DES

To check the performance of the catalyst to find the appropriate solvent (H_2_O, EtOH, H_2_O/EtOH, EtOAc, n-hexane, and solvent-free condition), temperature (50, 60, 70, 80, 90, and 100 °C), and amount of the catalyst (0.25, 0.5, 0.75, 1.0, 1.25, and 1.5 mol), the reaction between 4-chloro-benzaldehyde **(1)**, BA **(2)**, urea **(3)**, and was chosen as a model reaction for the synthesis of **4h** (Scheme 5).

The best result was found to be the 1:1:1 molar ratio of BA, urea, and 4-chloro-benzaldehyde with 1.0 mmol of the novel DES catalyst at 70 °C in solvent-free condition (Table 1).

**Table 1:** Optimization of the reaction conditions

Entry	Catalyst (mmol)	Temp. (°C)	Solvent	Yield (%)
1	0.25	70	–	87
2	0.50	70	–	91
3	0.75	70	–	93
4	1.25	70	–	90
5	1.50	70	–	91
6	1.0	50	–	90
7	1.0	60	–	89
8	1.0	80	–	86
9	1.0	90	–	83
10	1.0	100	–	76
11	1.0	Reflux	EtOH	85
12	1.0	Reflux	H_2_O/EtOH	69
13	1.0	Reflux	H_2_O	52
14	1.0	Reflux	*n*-Hexane	71
15	1.0	Reflux	EtOAc	82
16	1.0	70	–	92

#### Synthesis of 4(a–p)

Based on the obtained results from the model reaction, pyrimido[4,5-*d*]pyrimidines were synthesized under optimal reaction condition (Table 2). Short reaction times and high yields are important features of the proposed method.

**Table 2:** Synthesis of **4(a-p)** by DES


Entry	Aldehyde	Product	Time (min)	Yield (%)	M.P. °C Found, literature	TON	TOF
1		**4a**	35	93	228–230, NEW	93	2.65
2		**4b**	40	86	296–300, NEW	86	2.15
3		**4c**	35	94	217–220, NEW	94	2.68
4		**4d**	35	95	257–259, NEW	95	2.71
5		**4e**	45	79	223–225, NEW	79	1.75
6		**4f**	40	88	245–248, 241–244 [40]	88	2.20
7		**4g**	60	85	275–278, 282–284 [41]	85	1.41
8		**4h**	25	92	297–300, 292–295 [40]	92	3.68
9		**4i**	50	87	240–243, 239–240 [40]	87	1.74
10		**4j**	40	83	247–250, 199–202 [40]	83	2.07
11		**4k**	45	81	236–239, 221–224 [40]	81	1.80
12		**4l**	50	87	264–267, 251–254 [40]	87	1.74
13		**4m**	40	83	296–299, 284–287 [40]	83	2.07
14		**4n**	25	80	282–285, 271–273 [42]	80	3.20
15		**4o**	30	89	282–286 272–274 [43]	89	2.96
16		**4p**	40	75	221–224 215–216 [44]	75	1.87

#### Proposed mechanism for the synthesis of 4(a–p)

**Scheme 6:**
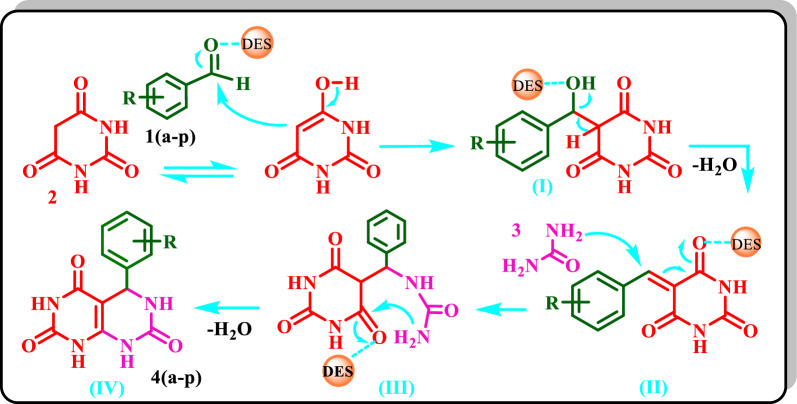
Proposed mechanism for the synthesis of **4(a**–**p)**

The possible mechanism for the synthesis of **4(a**–**p)** is shown in Scheme 6. First, the carbonyl group of an aldehyde is activated by the DES catalyst to be susceptible to the nucleophilic attack of BA to form (I). By removing water, **(II)** will be formed, and its condensation with urea yields **(III)**. Then, by internal cyclization of **(III)** and removing water, the final product **(IV)** is formed.

#### Reusability of DES in synthesis of 4(a–p)

**Fig. 7:**
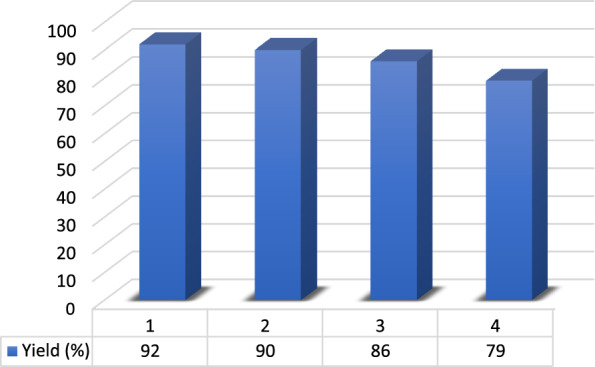
Reusability of DES

Catalyst reusability and recovery is an essential parameter to be considered. Therefore, after completion of the reaction (TLC) under optimal condition, it was stopped, and the resulting mixture was washed with ethanol to separate the catalyst. Ethanol was removed from the filtrate and the separated DES was dried and used in further four reaction runs. The efficiency of reactions was about 92, 90, 86 and 79%, respectively which confirms the stability of the prepared DES catalyst (Fig. 7).

#### Comparison of the catalyst activities

Table 3 shows the comparison of different methods for the synthesis of **4(a**–**p)**. Green condition, short reaction time, low temperature, easy separation of the DES catalyst and high efficiency are the advantages of our proposed method.

**Table 3:** Comparison of DES with the other catalysts

Entry	Catalyst	Condition	Time (min)	Yield (%)	Ref
1	MnCoFe_2_O_4_@ovalbumin	Solvent-free, 80 °C	10–25	80–96	[40]
2	CoFe_2_O_4_/TMU‐17‐NH_2_	Solvent-free, 80 °C	30	95–98	[41]
3	Catalyst free	Refluxing methanol	720	75–80	[42]
4	β-CD/H_2_O	60–65 °C	–	76–89	[43]
5	(α-Fe_2_O_3_)-MCM-41-DAIL	Solvent-free, 120 °C	180–480	91–98	[45]
6	CuI NPs	H_2_O, r.t	240–360	91–98	[46]
7	[H_2_-DABCO][ClO_4_]_2_	H_2_O, 75 °C	15–55	85–95	[47]
8	MTPP-Br/PHTH-DES	Solvent-free, 60 °C	43	85–95	Our work

### Optimization of the reaction conditions for the synthesis of 7c

**Scheme 7:**
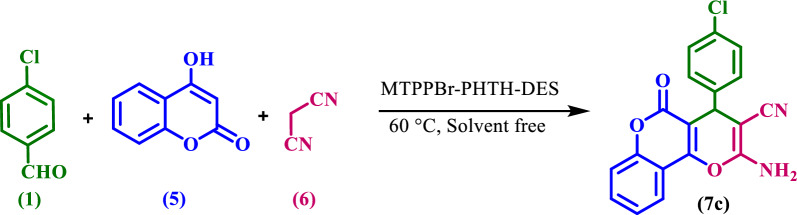
Synthesis of **7c** by DES

To check the performance of the catalyst to find an appropriate solvent (H_2_O, EtOH, H_2_O/EtOH, EtOAc, n-hexane, and solvent-free condition), temperature (50, 60, 70, 80, 90, and 100 °C), and amount of the catalyst (0.25, 0.5, 0.75, 1.0 1.25, and 1.5 mol), the reaction between 4-chlorobenzaldehyde **(1)**, 4-hydroxy-coumarin **(5)**, and malononitrile **(6)**, was chosen as a model reaction for the synthesis of **3c** (Scheme 7).

The best result was found to be the 1:1:1 mol ratio of BA, urea, and 4-chloro-benzaldehyde with 1.0 mmol of the novel DES catalyst at 60 °C in solvent-free condition (Table 4).

**Table 4:** Optimization of the reaction conditions

Entry	Catalyst (mmol)	Temp. (°C)	Solvent	Yield (%)
1	0.25	60	–	89
2	0.50	60	–	90
3	0.75	60	–	91
4	1.25	60	–	94
5	1.50	60	–	89
6	1.0	50	–	95
7	1.0	70	–	94
8	1.0	80	–	86
9	1.0	90	–	79
10	1.0	100	–	76
11	1.0	Reflux	EtOH	86
12	1.0	Reflux	H_2_O/EtOH	76
13	1.0	Reflux	H_2_O	70
14	1.0	Reflux	CH_3_CN	65
15	1.0	Reflux	CH_2_Cl_2_	42
16	1.0	60	–	96

### Synthesis of 7(a–j)

Table 5 shows the synthesis of diverse pyrano[3,2-*c*]chromenes under optimal condition in short reaction times and high yields.

**Table 5:** Synthesis of **7(a**–**j)** by MTPPBr-PHTH-DES


Entry	Aldehyde	Product	Time (min)	Yield (%)	M.P. °C Found, literature	TON	TOF
1		**7a**	10	95	262-265, 256-257 [48]	95	9.50
2		**7b**	15	97	263-267, 266–268 [49]	97	6.46
3		**7c**	10	96	259-262, 264-267 [49]	96	9.60
4		**7d**	20	93	260-263, 256-258 [49]	93	4.65
5		**7e**	10	92	256-258, 261-263 [49]	92	9.20
6		**7f**	10	90	273-275, 259-261 [49]	90	9.00
7		**7g**	20	91	234-236, 230-233 [50]	91	4.55
8		**7h**	25	85	230-233, 233-236 [50]	85	3.40
9		**7i**	20	88	248-252, 260–263 [51]	88	4.40
10		**7j**	25	89	220-224, 226-230 [48]	89	3.56

#### Proposed mechanism for the synthesis of pyrano[3,2-*c*]chromenes

**Scheme 8:**
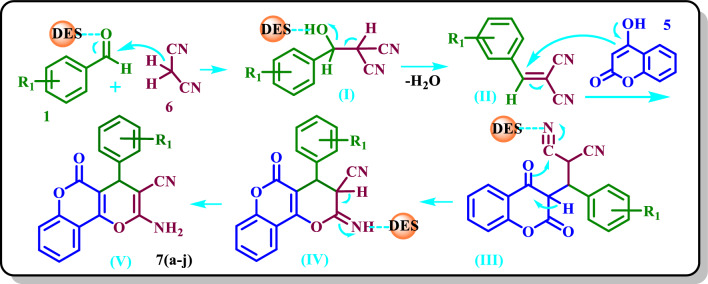
Proposed mechanism for the synthesis of **7(a**–**j)**

The possible mechanism for the synthesis of **7(a**–**j)** is shown in Scheme 8. First, the carbonyl group of an aldehyde is activated by the DES catalyst to be susceptible to the nucleophilic attack of malononitrile to form **(I)**. By removing water, **(II)** will be formed which condenses with 4-hydroxy-coumarin to yield **(III)**. Then, by internal cyclization of **(III)**, **(IV)** is formed, and the final product **(V)** will be formed by rearrangement of **(IV)**.

#### Reusability of DES in the synthesis of pyrano[3,2-*c*]chromenes

**Fig. 8:**
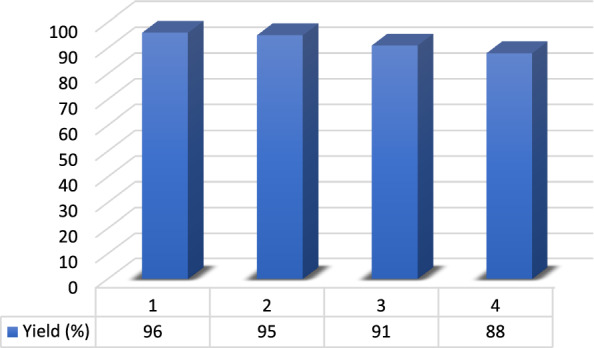
Reusability of DES

After completion of the reaction (TLC) under optimal condition, it was stopped, and the resulting mixture was washed with ethanol to separate the catalyst. Ethanol was removed from the filtrate and the separated DES was dried and used in further four reaction runs. The efficiency of reactions was about 96, 95, 91 and 88%, respectively which confirms the stability of the prepared DES catalyst (Fig. 8).

#### Comparison of the catalyst activities

Table 6 shows the comparison of different methods for the synthesis of **7(a**–**j)**. Green condition, short reaction time, low temperature, easy separation of the DES catalyst and high efficiency are the advantages of our proposed method.

**Table 6:** Comparison of DES with the other catalysts

Entry	Catalyst	Condition	Time (min)	Yield (%)	Refs.
1	SBPPSP	H_2_O/EtOH, Reflux	8–12	90–95	[49]
2	TBAB	Water	45–62	85–93	[50]
3	[TETA]TFA	H_2_O/EtOH, Reflux	5–240	50–94	[51]
4	Nano Al_2_O_3_	EtOH	300	75–89	[52]
5	TRUE	Water	8–25	88–94	[53]
6	DBU	Water/Reflux	5–20	81–94	[54]
7	H_5_BW_12_O_40_	H_2_O/EtOH, Reflux	195–270	85–98	[55]
8	DABCO	Neat	30	87–97	[56]
9	(CH_2_)_6_N_4_	EtOH, Reflux	7–80	89–95	[57]
10	DES	Solvent-free, 60 °C	10–25	85–97	Our work

## Experimental section

### Materials and methods

All chemicals were provided by the foreign chemical companies and used as received. Progress of the reactions was monitored by the TLC-silica gel 60 F-254 plates. The Fourier Transform Infrared (FT-IR) spectra were recorded by the Perkin-Elmer Spectrum Version 10.02.00 using KBr pellets. The NMR spectra were recorded on a 250 MHz Bruker spectrometer. Melting points were determined on a BUCHI 510 melting point apparatus. The density of the DES catalyst was determined by the AND-HR200 instrument. Thermo-Gravimetric-Analysis Differential-Thermal-Analysis (TGA-DTA) was done by the SDT-Q600 device.

#### General procedure for preparation of MTPPBr-PHTH-DES

The mixture of MTPPBr and PHTH (molar ratio 1:1) was stirred at 60 °C in solvent-free condition until a homogeneous liquid was obtained. When it was cooled slowly at room temperature, it turned into a transparent solid (DES) which dissolves well in water or ethanol.

#### General procedure for the synthesis of pyrimido[4,5-*d*]pyrimidines 4(a–p)

BA (1 mmol, 128 mg), urea (1 mmol, 60.06 mg), aldehyde (1.0 mmol), and DES catalyst (1 mmol, 0.523 g) were mixed and stirred at 70 °C in solvent‐free condition for an appropriate time. After completion of the reaction (TLC), the resulting mixture was washed with ethanol to separate the catalyst (the DES catalyst is soluble in ethanol and the reaction mixture is insoluble). Ethanol was removed from the filtrate and the separated DES was kept for further reactions.

A solid precipitate was washed several times with ethanol and characterized with comparison of their FT-IR, ^1^ HNMR, ^13^C NMR, Mass spectra, and melting points with authentic samples.

#### Spectral data of the 4(a–p)

##### 5-(4-Isopropylphenyl)-5,8-dihydropyrimido[4,5-*d*]pyrimidine-2,4,7(1*H*,3*H*,6*H*)-trione (4a)

Yellow solid, M.P.: 228–230 °C; IR (KBr) ν = 3202, 3089, 2957, 2868, 1751, 1703, 1673, 1578, 1440, 1414, 1343, 1307, 1212, 1196, 1136, 1077, 1045, 1021, 837, 813, 794, 634 and 545 cm^−1^. ^1^H NMR (250 MHz, DMSO-*d*_*6*_) δ = 11.34 (s, 1H), 11.19 (s, 1H), 8.24 (s, 1H), 7.72 (dd, *J* = 187.4, 7.9 Hz, 5H), 5.47 (s, 1H). 2.93 (h, *J* = 6.7 Hz, 1H), 1.21 (d, *J* = 6.8 Hz, 6H). ^13^C NMR (62.5 MHz, DMSO-*d*_*6*_) δ = 164.0, 162.2, 155.3, 154.3, 150.6, 134.5, 133.7, 130.7, 126.6, 125.6, 124.8, 118.3, 34.0, 32.0, 26.6, 23.8.

##### 4-(2,5,7-Trioxo-1,2,3,4,5,6,7,8-octahydropyrimido[4,5-*d*]pyrimidin-4-yl)benzoic acid (4b)

Yellow solid, M.P.: 296–300 °C; IR (KBr) ν = 3271, 3247, 3131, 2874, 1777, 1766, 1734, 1705, 1599, 1417, 1366, 1322, 1289, 1252, 1218, 1059, 1041, 966, 772, 744, 685 and 502 cm^−1^. ^1^H NMR (250 MHz, DMSO-*d*_*6*_) δ = 11.57 (s, 1H), 11.47–11.18 (m, 2H), 10.98–10.81 (m, 2H), 7.85–7.81 (m, 2H), 7.73–7.69 (m, 1H), 7.54–7.52 (m, 1H), 6.23 (s, 1H). ^13^C NMR (62.5 MHz, DMSO-*d*_*6*_) δ = 169.5, 162.7, 150.8, 134.5, 134.4, 133.3, 129.4, 129.4, 127.5, 125.5, 123.2, 78.9, 51.1. MS: m/z = 302.1 [M]^+^, Base peak: m/z = 231.2.

##### 5-(4-(Diethylamino)phenyl)-5,8-dihydropyrimido[4,5-*d*]pyrimidine-2,4,7(1*H*,3*H*,6*H*)-trione (4c)

Yellow solid, M.P.: 217–220 °C; IR (KBr) ν = 3445, 3271, 3208, 3027, 1736, 1684, 1652, 1608, 1449, 1443, 1391, 1345, 1311, 1187, 1153, 1075, 1011, 795, 677 and 518 cm^−1^. ^1^H NMR (250 MHz, DMSO-*d*_*6*_) δ = 11.04 (s, 1H), 10.91 (s, 1H), 8.41 (s, 1H), 8.11 (s, 1H), 7.80–5.86 (m, 4H), 5.44 (s, 1H), 3.49–3.42 (m, 4H), 1.12 (t, *J* = 7.6 Hz, 6H).^13^C NMR (62.5 MHz, DMSO-*d*_*6*_) δ = 165.2, 165.2, 163.2, 160.1, 155.7, 155.7, 152.6, 150.8, 142.8, 139.9, 120.1, 111.3, 111.3, 109.3, 44.6, 12.9.; MS: m/z = 329.2 [M]^+^, Base peak: m/z = 272.1.

##### 5-(3-Ethoxy-4-hydroxyphenyl)-5,8-dihydropyrimido[4,5-*d*]pyrimidine2,4,7(1*H*,3*H*,6*H*)-trione (4d)

Yellow solid, M.P.: 257–259 °C; IR (KBr) ν = 3512, 3205, 3039, 2842, 1761, 1701, 1652, 1543, 1505,1405, 1349, 1277, 981, 793, 753, 513 and 407 cm^−1^. ^1^H NMR (250 MHz, DMSO-*d*_*6*_) δ = 11.22 (d, *J* = 6.9 Hz, 1H), 11.10 (d, *J* = 6.8 Hz, 1H), 10.45 (d, *J* = 7.3 Hz, 1H), 8.46 (d, *J* = 5.9 Hz, 1H), 8.18 (d, *J* = 7.6 Hz, 1H), 7.76–6.83 (m, 3H), 5.39 (s, 1H), 4.09–4.03 (m, 2H), 1.38–1.32 (m, 3H). ^13^C NMR (62.5 MHz, DMSO-*d*_*6*_) δ = 165.1, 164.6, 162.9, 156.4, 153.7, 150.6, 146.5, 132.9, 124.6, 119.4, 115.8, 114.3, 64.2, 15.0.; MS: m/z = 318.2 [M]^+^, Base peak: m/z = 276.1.

##### 5-(2,3-Dihydroxyphenyl)-5,8-dihydropyrimido[4,5-*d*]pyrimidine-2,4,7(1*H*,3*H*,6*H*)-trione (4e)

Yellow solid, M.P.: 223–225 °C; IR (KBr) ν = 3600, 3438, 3361, 3223, 3096, 1748, 1712, 1635, 1474, 1456, 1347, 1216, 1149, 845, 781, 496 and 419 cm^−1^. ^1^H NMR (250 MHz, DMSO-*d*_*6*_) δ = 11.89 (s, 1H), 11.24 (s, 1H), 11.15–11.08 (m, 1H), 10.95 (s, 1H), 10.12–10.00 (m, 1H), 7.00–6.79 (m, 2H), 6.50 (d, J = 7.3 Hz, 1H), 5.42 (s, 1H), 4.64 (s, 1H). ^13^C NMR (62.5 MHz, DMSO-*d*_*6*_) δ = 164.0, 160.1, 151.0, 149.9, 145.9, 138.4, 125.6, 122.2, 117.7, 116.1, 85.5, 53.5.; MS: m/z = 290.1 [M]^+^, Base peak: m/z = 230.

##### 5-Phenyl-5,8-dihydropyrimido[4,5-*d*]pyrimidine-2,4,7(1*H*,3*H*,6*H*)-trione (4f)

Yellow solid, M.P.: 262–265 °C; IR (KBr) ν = 3215, 3069, 2836, 1743, 1677, 1582, 1566, 1451, 1405, 1341, 1297, 1203, 1033, 864, 763, 680, 526 and 419 cm^−1^.

##### 5-(2-Chlorophenyl)-5,8-dihydropyrimido[4,5-*d*]pyrimidine-2,4,7(1*H*,3*H*,6*H*)-trione (4g)

Yellow solid, M.P.: 215–218 °C; IR (KBr) ν = 3487, 3450, 3210, 3051, 2819, 1766, 1678, 1611, 1431, 1383, 1275, 1228, 1049, 943, 806, 789, 754, 609, 525, 505, and 435 cm^−1^.

##### 5-(4-Chlorophenyl)-5,8-dihydropyrimido[4,5-*d*]pyrimidine-2,4,7(1*H*,3*H*,6*H*)-trione (4h)

Yellow solid, M.P.: 297–300 °C; IR (KBr) ν = 3212, 3087, 2845, 1755, 1704, 1673, 1574, 1554, 1443, 1413, 1344, 1289, 1202, 1092, 1019, 882, 838, 809, 793, 550 and 423 cm^−1^.

##### 5-(2,4-Dichlorophenyl)-5,8-dihydropyrimido[4,5-*d*]pyrimidine-2,4,7(1*H*,3*H*,6*H*)-trione (4i)

Yellow solid, M.P.: 270–273 °C; IR (KBr) ν = 3211, 3074, 2832, 1759, 1722, 1691, 1578, 1439, 1379, 791 and 509 cm^−1^.

##### 5-(3-Nitrophenyl)-5,8-dihydropyrimido[4,5-*d*]pyrimidine-2,4,7(1*H*,3*H*,6*H*)-trione (4j)

Yellow solid, M.P.: 247–250 °C; IR (KBr) ν = 3409, 3083, 2963, 2778, 1755, 1725, 1688, 1650, 1526, 1409, 1380, 1352, 1246, 1218, 1080, 1009, 909, 843, 809, 728, 499 and 417 cm^−1^.

##### 5-(4-Nitrophenyl)-5,8-dihydropyrimido[4,5-*d*]pyrimidine-2,4,7(1*H*,3*H*,6*H*)-trione (4k)

Yellow solid, M.P.: 246–249 °C; IR (KBr) ν = 3343, 3269, 3078, 2995, 2837, 1721, 1650, 1519, 1419, 1379, 1349, 1292, 1238, 1109, 1013, 860, 778, 697, 657, 550 and 534 cm^−1^.

##### 5-(*p*-Tolyl)-5,8-dihydropyrimido[4,5-*d*]pyrimidine-2,4,7(1*H*,3*H*,6*H*)-trione (4l)

Yellow solid, M.P.: 270–275 °C; IR (KBr) ν = 3467, 3210, 3081, 2839, 1752, 1675, 1556, 1430, 1344, 1296, 1192, 822, and 523 cm^−1^.

##### 5-(4-Methoxyphenyl)-5,8-dihydropyrimido[4,5-*d*]pyrimidine-2,4,7(1*H*,3*H*,6*H*)-trione (4m)

Yellow solid, M.P.: 297–300 °C; IR (KBr) ν = 3208, 3070, 2841, 1729, 1673, 1550, 1508, 1435, 1401, 1308, 1271, 1180, 1015, 837, 794, 628, 519 and 421 cm^−1^.

##### 5-(3,4-Dimethoxyphenyl)-5,8-dihydropyrimido[4,5-*d*]pyrimidine-2,4,7(1*H*,3*H*,6*H*)-trione (4n)

Yellow solid, M.P.: 320–324 °C; IR (KBr) ν = 3226, 3146, 3076, 2950, 1740, 1696, 1655, 1541, 1496, 1424, 1390, 1276, 1148, 1011, 798, 524 and 483 (cm^−1^).

##### 5-(Furan-2-yl)-5,8-dihydropyrimido[4,5-*d*]pyrimidine-2,4,7(1*H*,3*H*,6*H*)-trione (4o)

Yellow solid, M.P.: 323–325 °C; IR (KBr) ν = 3512, 3205, 3121, 3037, 2842, 1741, 1761, 1698, 1651, 1543, 1499, 1441, 1276, 1163, 972, 793, 753, and 515 (cm^−1^).

##### 5-(Thiophen-2-yl)-5,8-dihydropyrimido[4,5-*d*]pyrimidine-2,4,7(*1H*,*3H*,*6H*)-trione (4p)

Yellow solid, M.P.: 330–333 °C; IR (KBr) ν = 3226, 3146, 3076, 2950, 1740, 1696, 1655, 1541, 1496, 1424, 1390, 1276, 1148, 1011, 798, 524 and 483 (cm^−1^).

### General procedure for the synthesis of pyrano[3,2-*c*]chromenes 7(a–j)

4-Hydroxycoumarin (1 mmol, 162 mg), malononitrile (1 mmol, 66 mg), aldehyde (1.0 mmol), and the DES catalyst (1 mmol, 0.523 mg) were mixed and stirred at 70 °C in solvent‐free condition for an appropriate time. After completion of the reaction (TLC), the resulting mixture was washed with ethanol to separate the catalyst (the DES catalyst is soluble in ethanol and the reaction mixture is insoluble). Ethanol was removed from the filtrate and the separated DES was kept for further reactions.

A solid precipitate was washed several times with ethanol and characterized with comparison of their FT-IR, ^1^HNMR spectra, and melting points with authentic samples.

#### Spectral data of the 7(a–j)

##### 2-Amino-5-oxo-4-phenyl-4*H*,5*H*-pyrano[3,2-*c*]chromene-3-carbonitrile (7a)

White solid, M.P.: 228–230 °C; IR (KBr) ν = 3376, 3285, 3065, 2198, 1709, 1675, 1605, 1638, 1382, 1211, 1113, 1058, 956, 758, 706 and 522 (cm^−1^).

##### 2-Amino-4-(2-chlorophenyl)-5-oxo-4*H*,5*H*-pyrano[3,2-*c*]chromene-3-carbonitrile (7b)

White solid, M.P.: 228–230 °C; IR (KBr) ν = 3402, 3284, 3180, 3086, 2192, 1710, 1675, 1602, 1458, 1382, 1174, 1063, 958, 904, 756 and 522. (cm^−1^).

##### 2-Amino-4-(4-chlorophenyl)-5-oxo-4*H*,5*H*-pyrano[3,2-*c*]chromene-3-carbonitrile (7c)

White solid, M.P.: 228–230 °C; IR (KBr) ν = 3382, 3307, 3291, 2193, 1714, 1676, 1603, 1562, 1458, 1378, 1213, 1092, 1061, 906, 845, 765 and 513 (cm^−1^).

##### 2-Amino-4-(2,4-dichlorophenyl)-5-oxo-4*H*,5*H*-pyrano[3,2-*c*]chromene-3-carbonitrile (7d)

White solid, M.P.: 228–230 °C; IR (KBr) ν = 3323, 3204, 2195, 1720, 1668, 1601, 1512, 1381, 1264, 1143, 1095, 762 and 481 (cm^−1^).

##### 2-Amino-4-(3-nitrophenyl)-5-oxo-4*H*,5*H*-pyrano[3,2-*c*]chromene-3-carbonitrile (7e)

White solid, M.P.: 228–230 °C; IR (KBr) ν = 3322, 3193, 3093, 2994, 2203, 1703, 1672, 1606, 1531, 1381, 1347, 1176, 1058, 898, 762 and 709 (cm^−1^).

##### 2-Amino-4-(4-nitrophenyl)-5-oxo-4*H*,5*H*-pyrano[3,2-*c*]chromene-3-carbonitrile (7f)

White solid, M.P.: 228–230 °C; IR (KBr) ν = 3369, 3335, 3073, 2195, 1717, 1671, 1606, 1505, 1347, 1370, 1055, 765 and 459 (cm^−1^).

##### 2-Amino-4-(3,4-dimethoxyphenyl)-5-oxo-4*H*,5*H*-pyrano[3,2-*c*]chromene-3-carbonitrile (7g)

White solid, M.P.: 234–236 °C; IR (KBr) ν = 3406, 3326, 3261, 2196, 1709, 1673, 1378, 1048 and 760. ^1^H NMR (250 MHz, DMSO-*d*_*6*_) δ = 7.87 (d, J = 7.8 Hz, 1H), 7.68 (t, J = 7.8 Hz, 1H), 7.45 (t, J = 9.3 Hz, 2H), 7.35 (s, 2H), 6.85 (d, J = 12.4 Hz, 2H), 6.72 (d, J = 8.3 Hz, 1H), 4.38 (s, 1H), 3.69 (s, 6H). ^13^C NMR (62.5 MHz, DMSO-*d*_*6*_) δ = 158.4, 152.5, 149, 149, 136.3, 133.3, 125.1, 122.9, 120.1, 119.8, 117, 112.3, 112, 104.5, 58.5, 55.9, 40.9, 40.6, 40.3, 39.9, 39.6, 39.3, 38.9, 36.9.

##### 2-Amino-4-(4-isopropylphenyl)-5-oxo-4*H*,5*H*-pyrano[3,2-*c*]chromene-3-carbonitrile (7h)

White solid, M.P.: 230–233 °C; IR (KBr) ν = 3390, 3304, 3205, 2202, 1713, 1672, 1375, 1050 and 769. (cm^−1^).

##### 2-Amino-4-(4-hydroxyphenyl)-5-oxo-4*H*,5*H*-pyrano[3,2-*c*]chromene-3-carbonitrile (7i)

White solid, M.P.: 228–230 °C; IR (KBr) ν = 3504, 3410, 3287, 3184, 3068, 2879, 2197, 1697, 1674, 1610, 1513, 1460, 1382, 1266, 1071, 844, 760, 562 and 524 (cm^−1^).

##### 2-Amino-5-oxo-4-(thiophen-2-yl)-4*H*,5*H*-pyrano[3,2-*c*]chromene-3-carbonitrile (7j)

White solid, M.P.: 228–230 °C; IR (KBr) ν = 3368, 3279, 3177, 3070, 2200, 1709, 1668, 1601, 1564, 1308, 1056, 760 and 705 (cm^−1^).

## Conclusion

In conclusion, the new DES (MTPPBr-PHTH-DES) was prepared, characterized and used as a novel catalyst in the synthesis of pyrimido[4,5-*d*]pyrimidines **4(a**–**p)** and pyrano[3,2-*c*]chromenes **7(a**–**j)**. The newly synthesized DES catalyst can be synthesized simply by mixing and heating the starting materials, which does not require any additional purification steps, and a simple filtration is sufficient to separate it from the reaction mixture. It can be used not only as a moderate, inexpensive, and environmentally safe solvent, but also as recyclable and reusable organocatalyst to facilitate organic transformations.

### Supplementary Information

Supplementary Material 1.

## Data Availability

All the methods carried out in this project are in accordance with relevant local/national/ international institutional guidelines and regulations. All data generated or analyzed during this study are not publicly available due to DATA NOT PUBLIC but are available from the corresponding author on reasonable request.
