# Supplementary material for: The new phthalic acid-based deep eutectic solvent as a versatile catalyst for the synthesis of pyrimido[4,5-d]pyrimidines and pyrano[3,2-c]chromenes
Source: BMC Chem. 2024 Jun 27;18(1):120. doi: 10.1186/s13065-024-01227-x (PMC11212169; doi:10.1186/s13065-024-01227-x)

**Supporting Information**

**(BMC Chemistry)**

**The new phthalic acid-based deep eutectic solvent as a versatile catalyst for the synthesis of pyrimido[4,5-*d*]pyrimidines and pyrano[3,2-*c*]chromenes**

**Arezo Monem^1^, Davood Habibi^1^*, Abdolhamid Alizadeh^2^, Hadis Goudarzi^1^**

**^1^**Department of Organic Chemistry and Petroleum Chemistry, Faculty of Chemistry, Bu-Ali Sina University, Hamedan 6517838683, Iran

**^2^**Department of Organic Chemistry, Faculty of Chemistry, Alzahra University, Tehran 1993893973 Iran

*Corresponding author email: [davood.habibi@gmail.com](mailto:davood.habibi@gmail.com) (dhabibi@basu.ac.ir), Tel: +98 81 38380922; Fax: +98 81 31408025

**Content Page**

FT-IR spectrum of 4a 3

^1^H NMR spectrum of 4a 3

^13^C NMR spectrum of 4a 4

FT-IR spectrum of 4b 4

^1^H NMR spectrum of 4b 5

^13^C NMR spectrum of 4b 5

Mass spectrum of 4b 6

FT-IR spectrum of 4c 6

^1^H NMR spectrum of 4c 7

^13^C NMR spectrum of 4c 7

Mass spectrum of 4c 8

FT-IR spectrum of 4d 8

^1^H NMR spectrum of 4d 9

^13^C NMR spectrum of 4d 9

Mass spectrum of 4d 10

FT-IR spectrum of 4e 10

^1^H NMR spectrum of 4e 11

^13^C NMR spectrum of 4e 11

Mass spectrum of 4e 12

FT-IR spectrum of 4f 12

FT-IR spectrum of 4g 12

FT-IR spectrum of 4h 13

FT-IR spectrum of 4i 13

FT-IR spectrum of 4j 13

FT-IR spectrum of 4k 14

FT-IR spectrum of 4l 14

FT-IR spectrum of 4m 14

FT-IR spectrum of 4n 15

FT-IR spectrum of 4o 15

FT-IR spectrum of 4p 15

FT-IR spectrum of 7a 16

FT-IR spectrum of 7b 16

^1^H NMR spectrum of 7b 17

FT-IR spectrum of 7c 17

FT-IR spectrum of 7f 18

FT-IR spectrum of 7g 18

^1^H NMR spectrum of 7g 19

^13^C NMR spectrum of 7g 19

FT-IR spectrum of 7h 20

FT-IR spectrum of 7i 20

FT-IR spectrum of 7j 20

FT-IR spectrum of **2a**


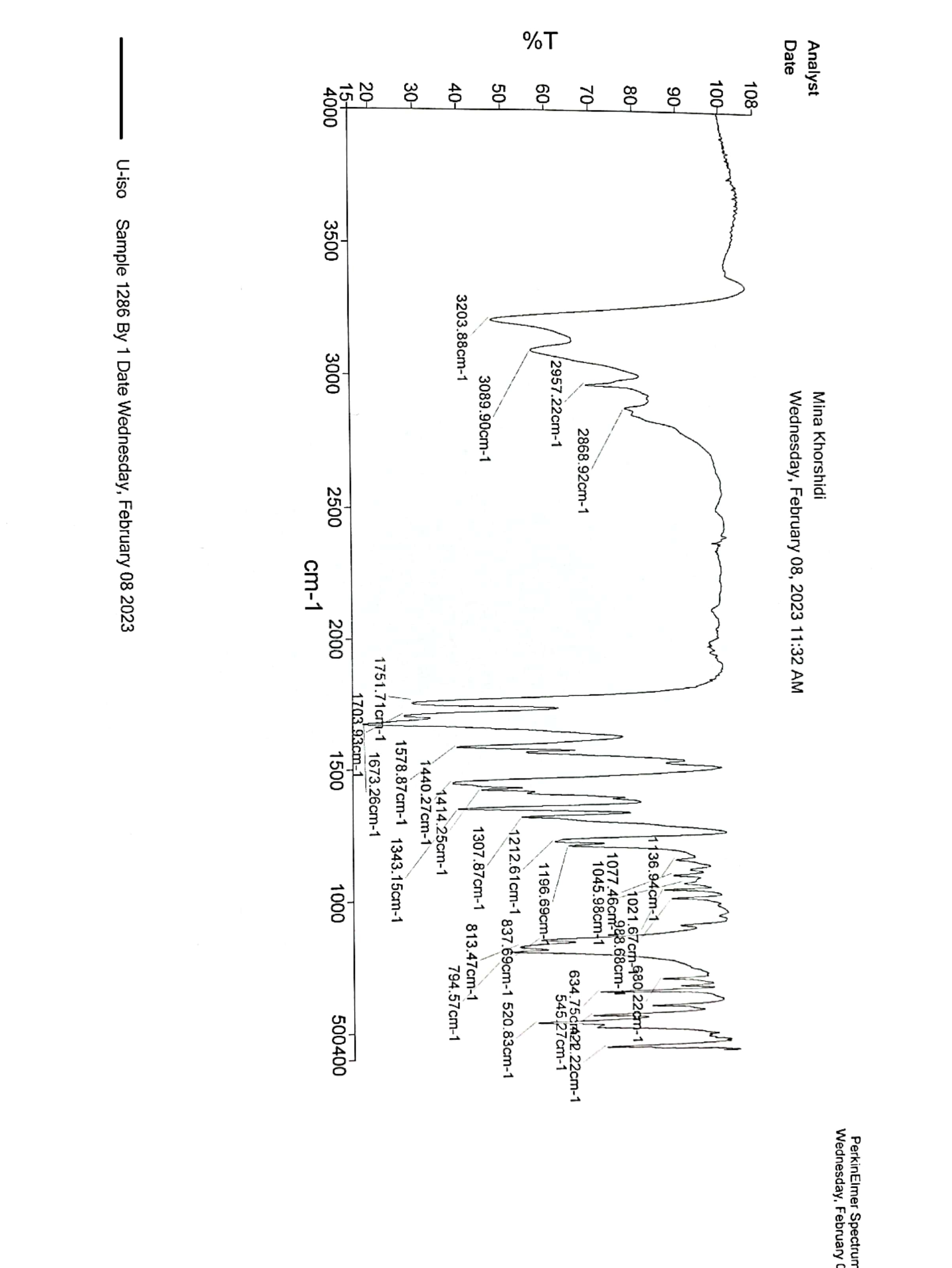


^1^H NMR spectrum of **2a**

^13^C NMR spectrum of **2a**

FT-IR spectrum of **2b**


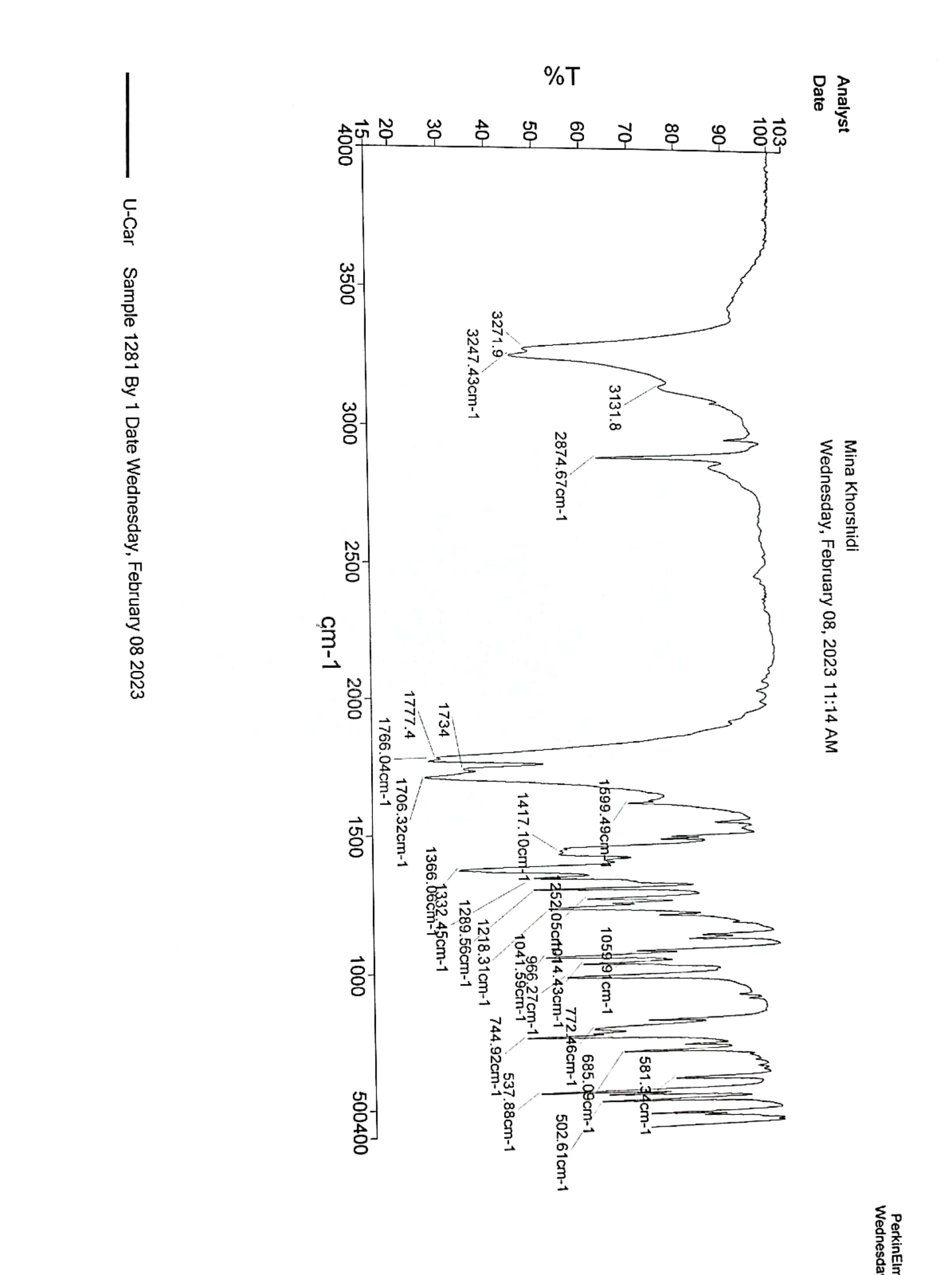


^1^H NMR spectrum of **2b**

^13^C NMR spectrum of **2b**

Mass spectrum of **2b**


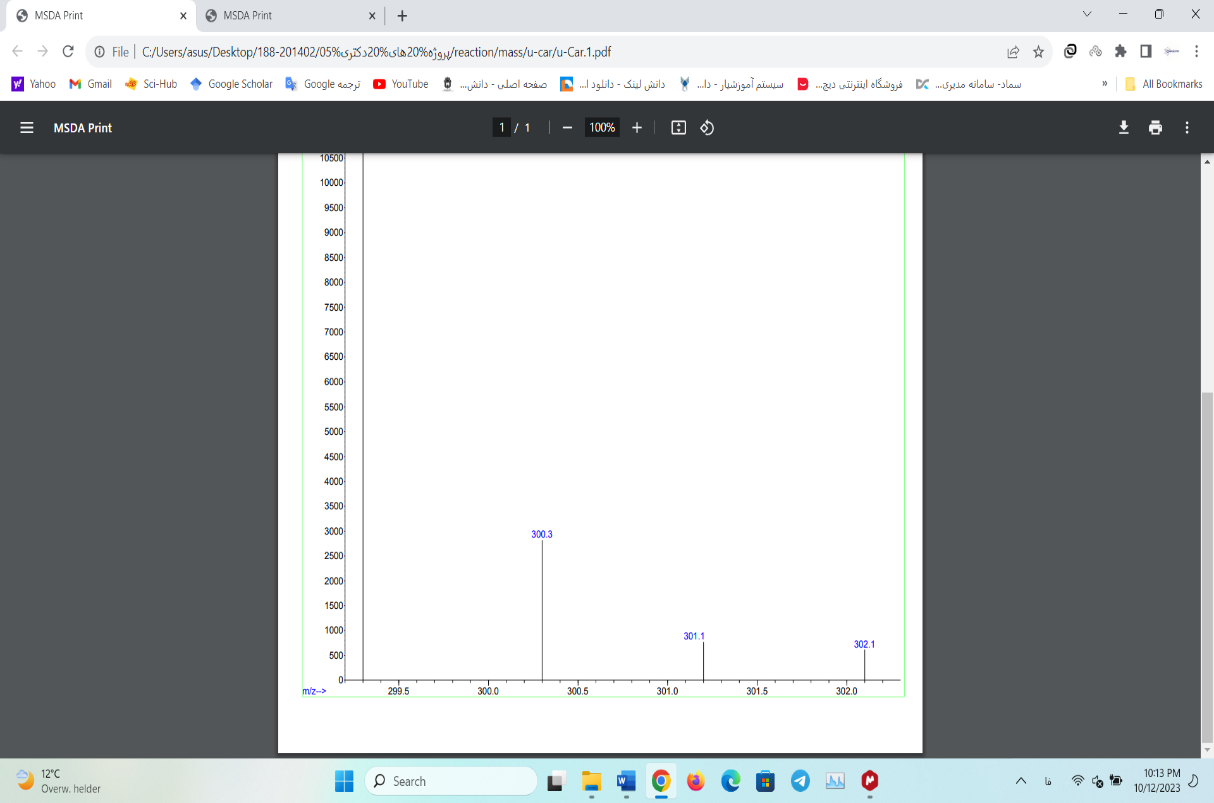

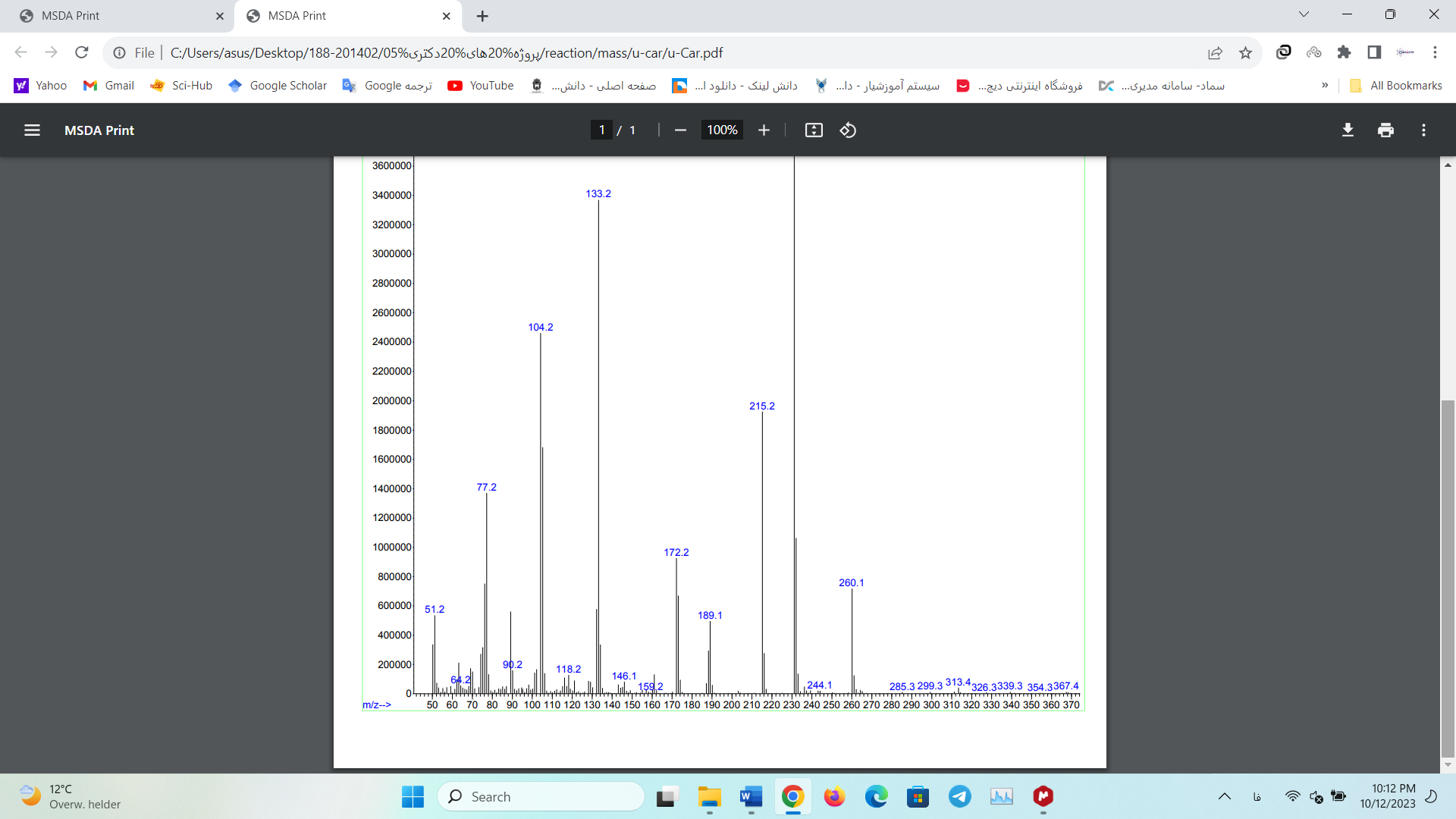


FT-IR spectrum of **2c**


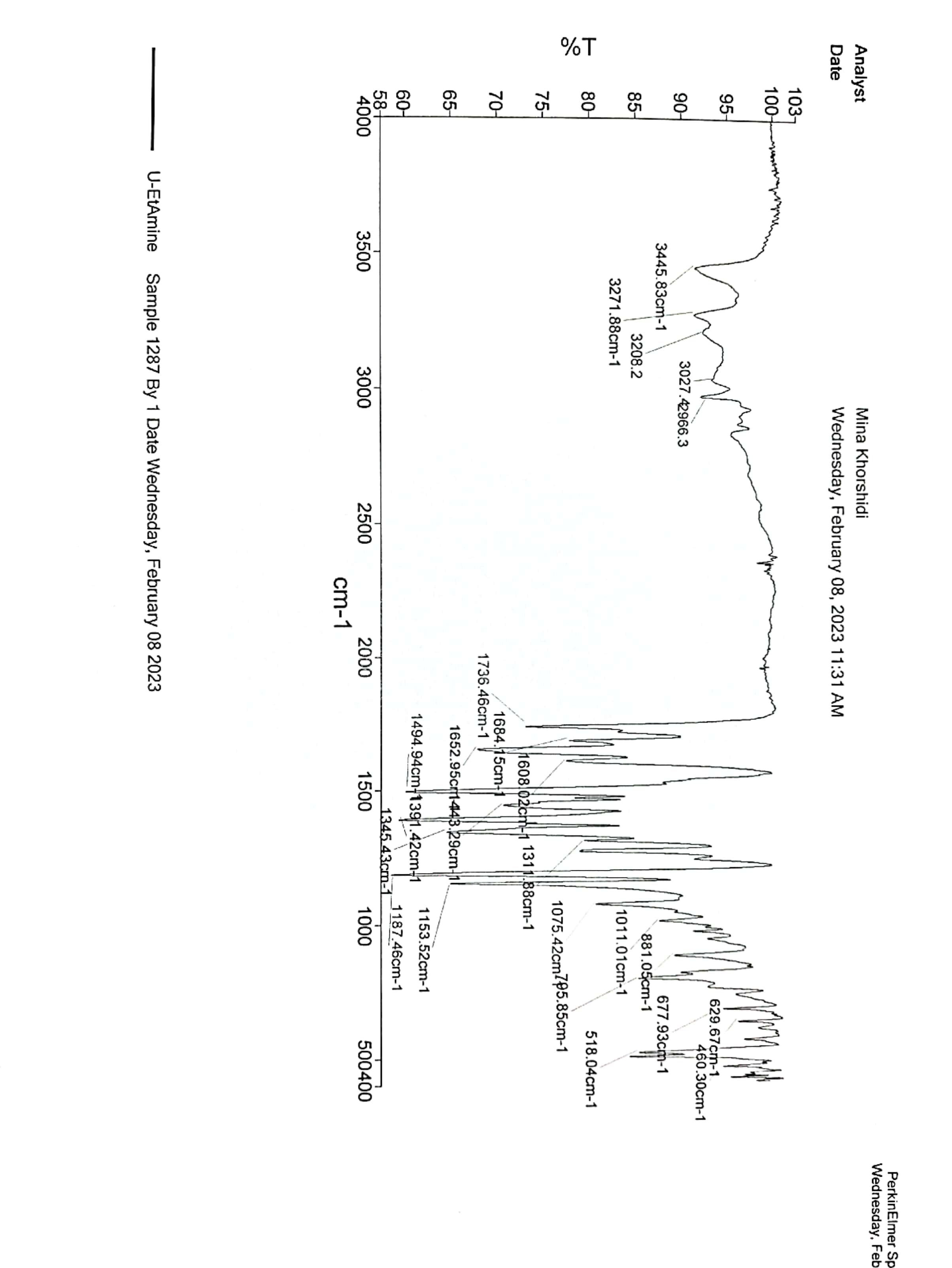


^1^H NMR spectrum of **2c**

^13^C NMR spectrum of **2c**

Mass spectrum of **2c**


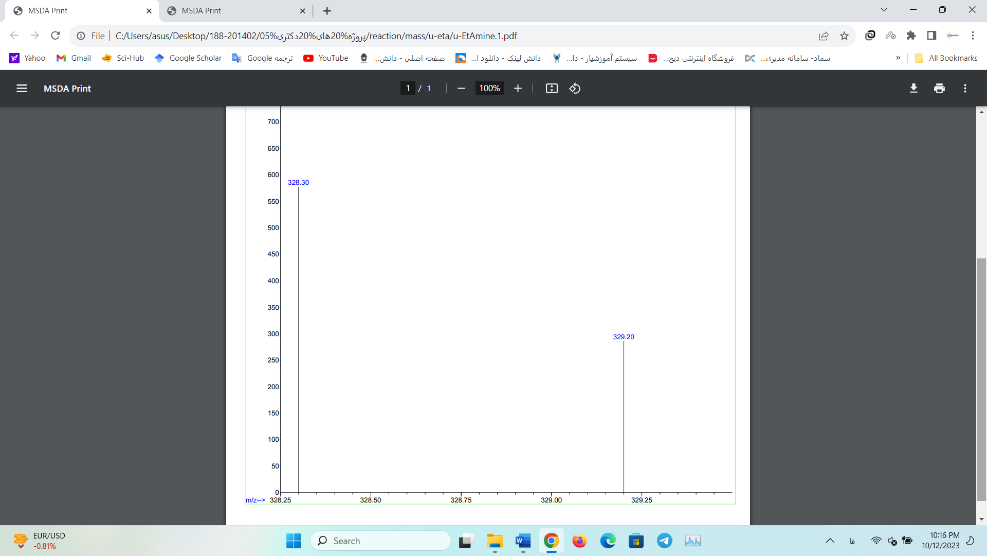

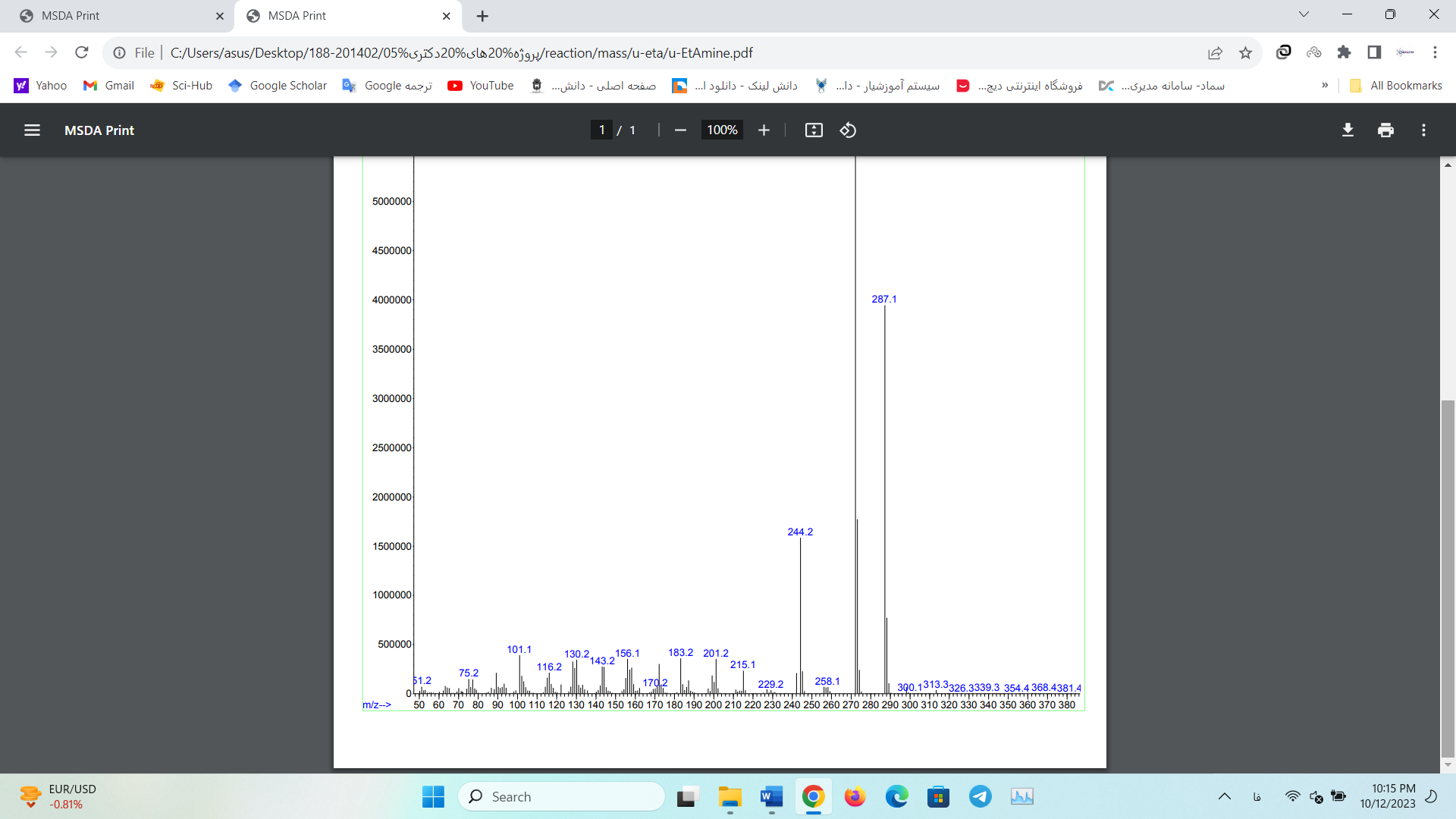


FT-IR spectrum of **2d**


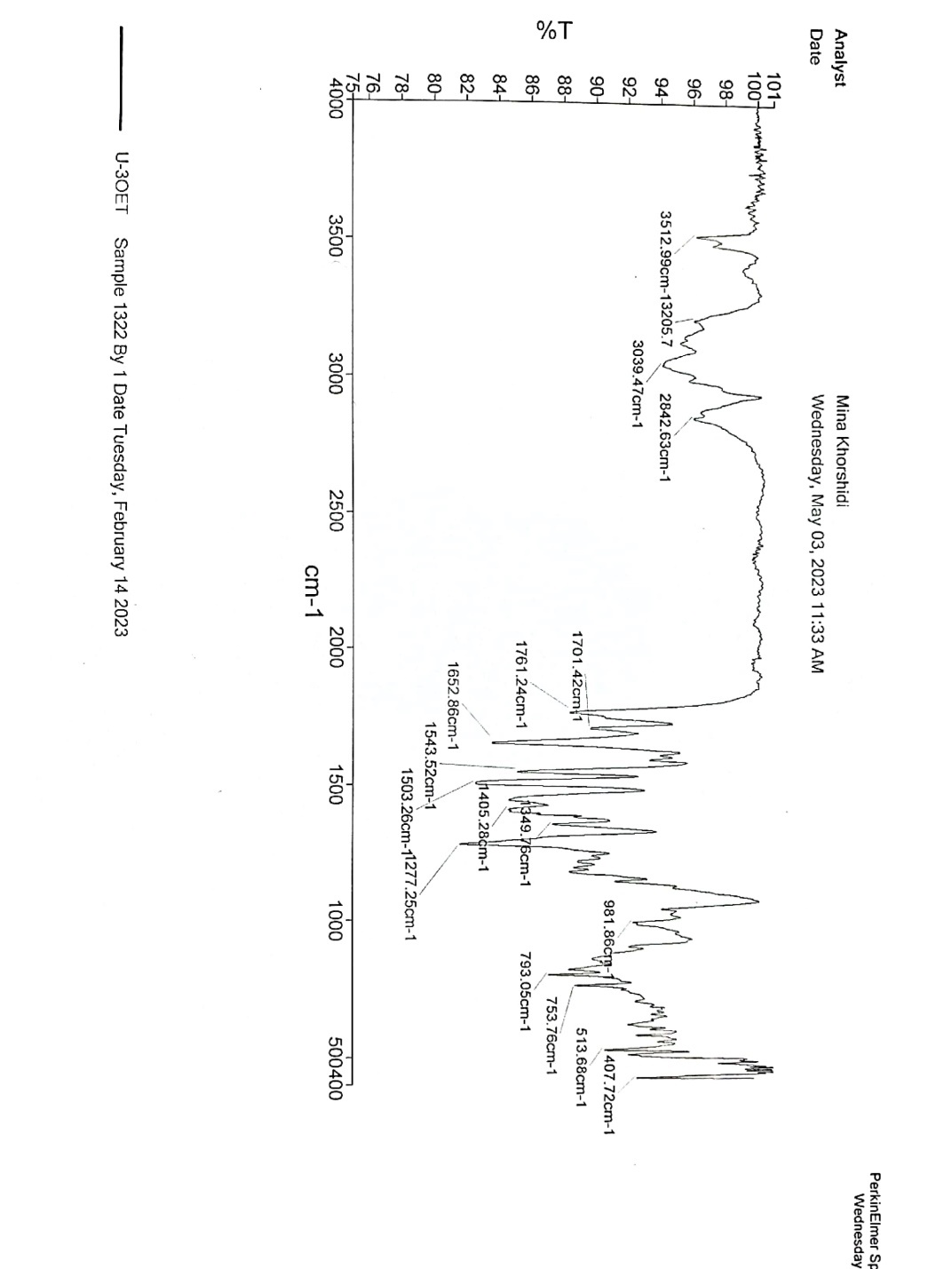


^1^H NMR spectrum of **2d**

^13^C NMR spectrum of **2d**

Mass spectrum of **2d**


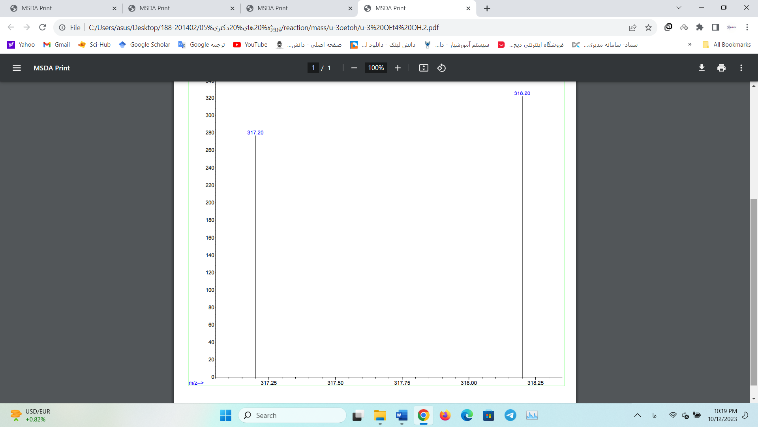

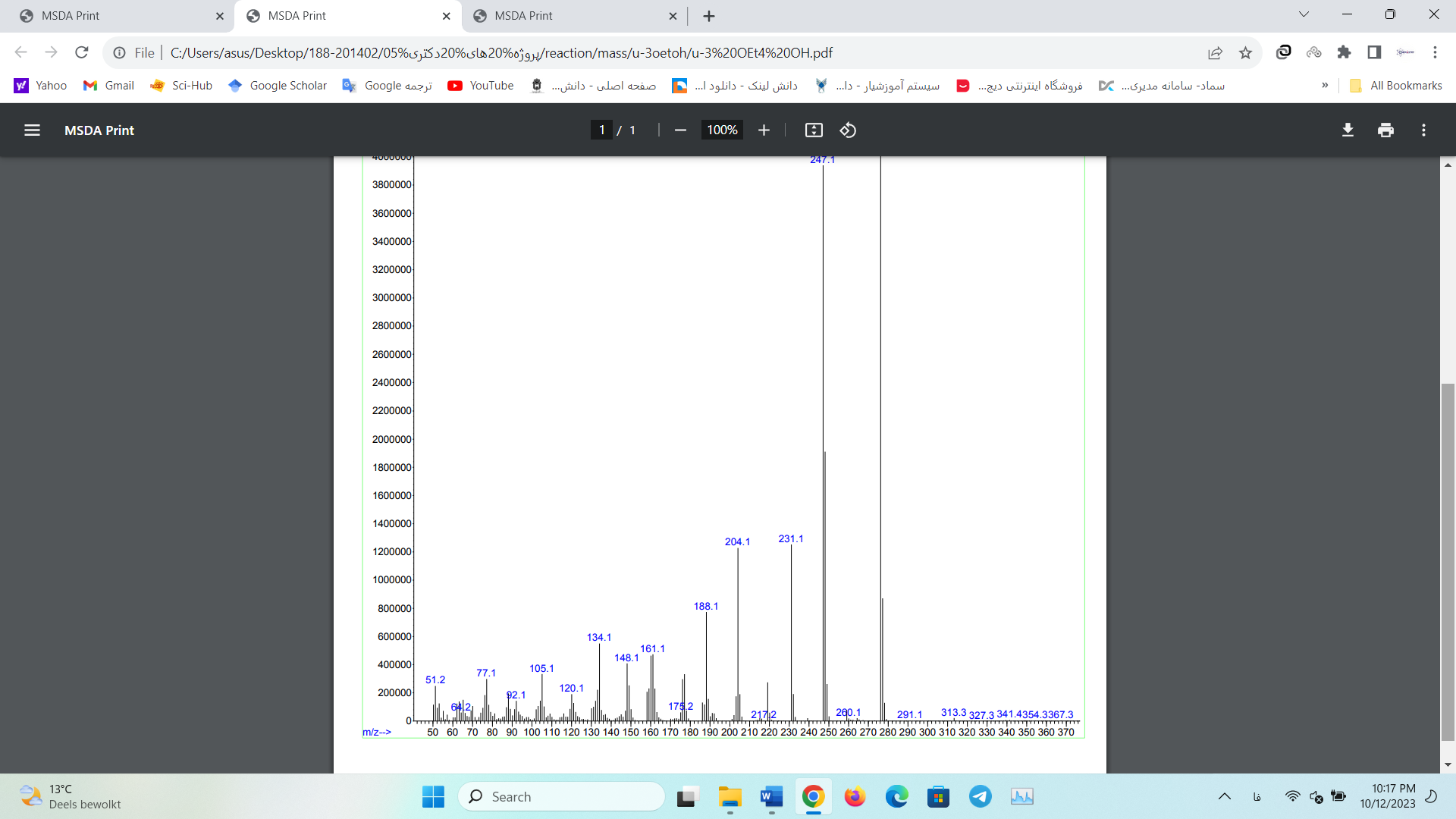


FT-IR spectrum of **2e**


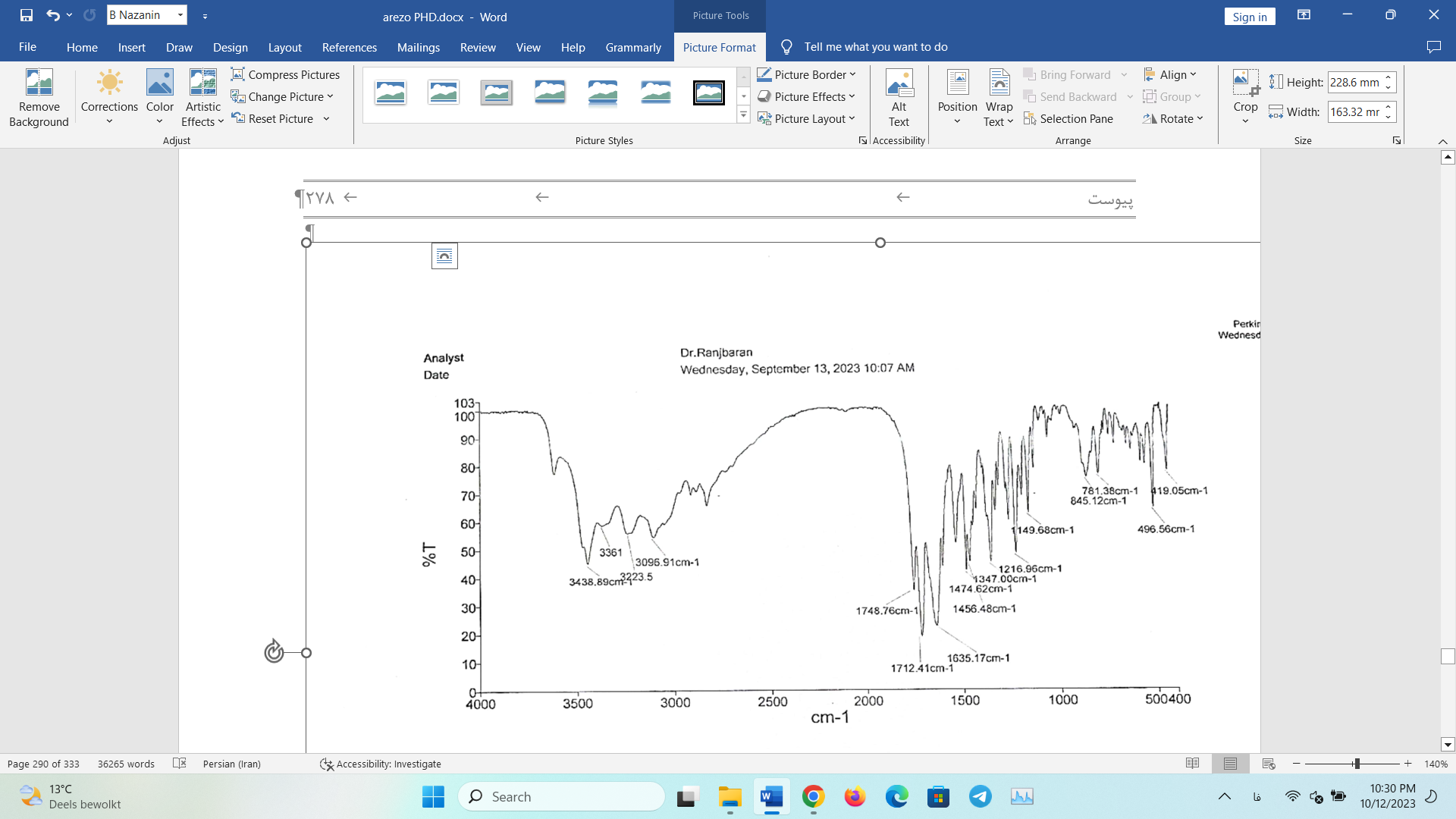


^1^H NMR spectrum of **2e**

^13^C NMR spectrum of **2e**

Mass spectrum of **2e**


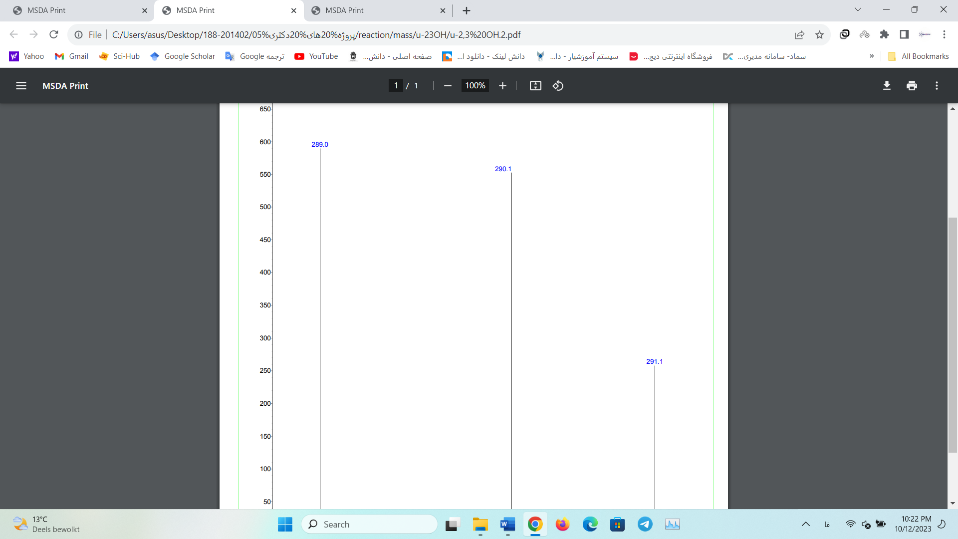

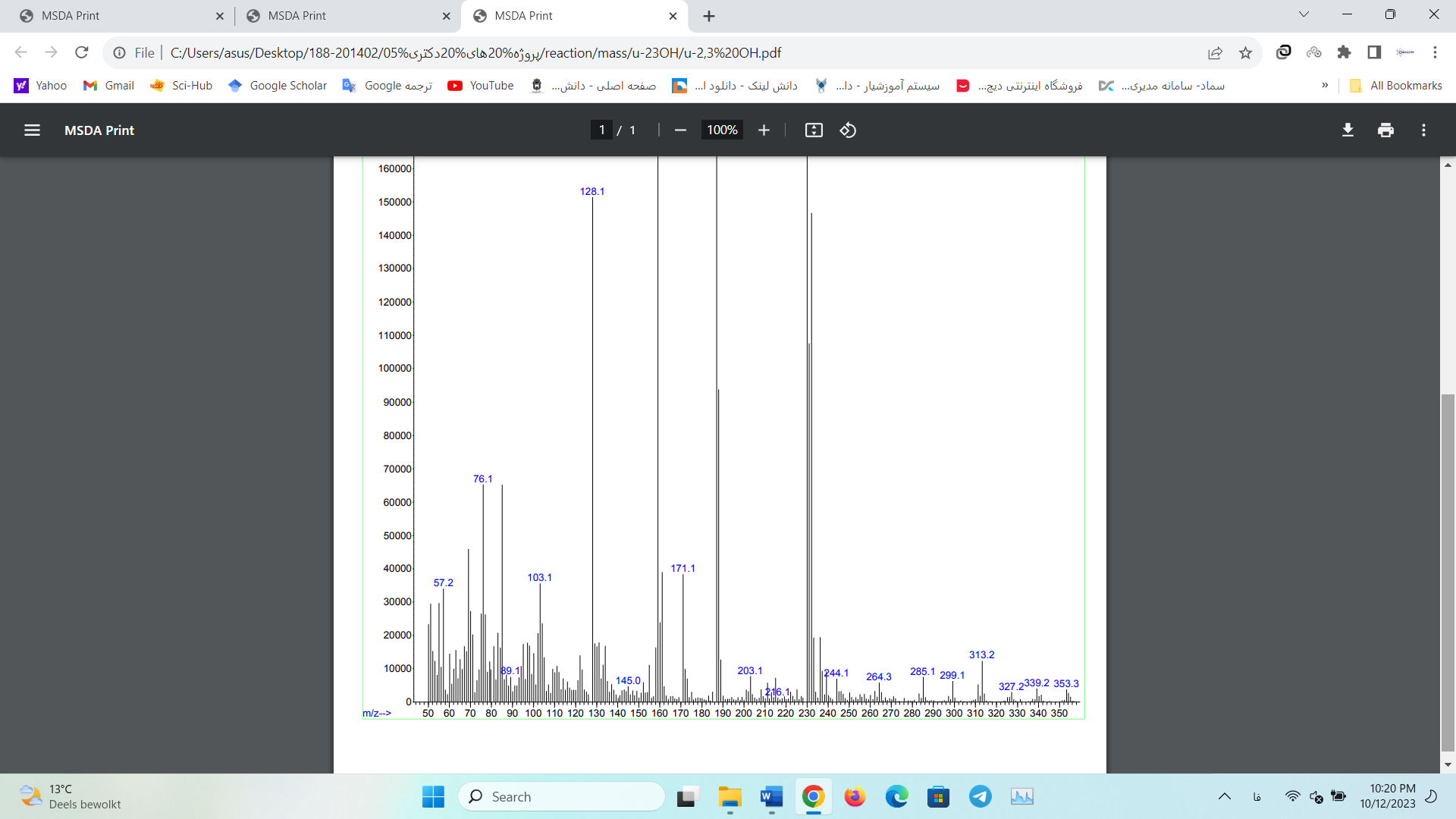


FT-IR spectrum of **2f**


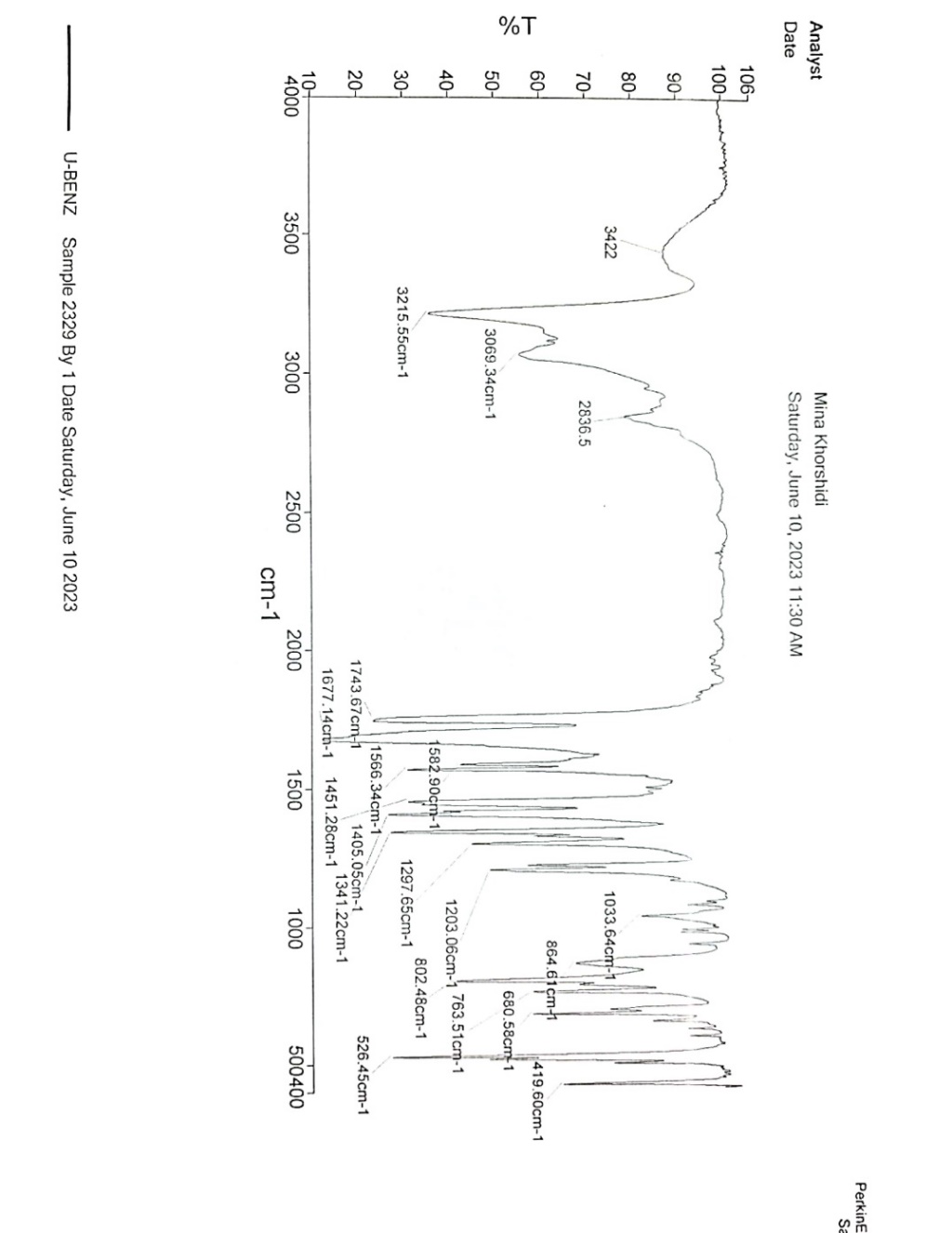


FT-IR spectrum of **2g**


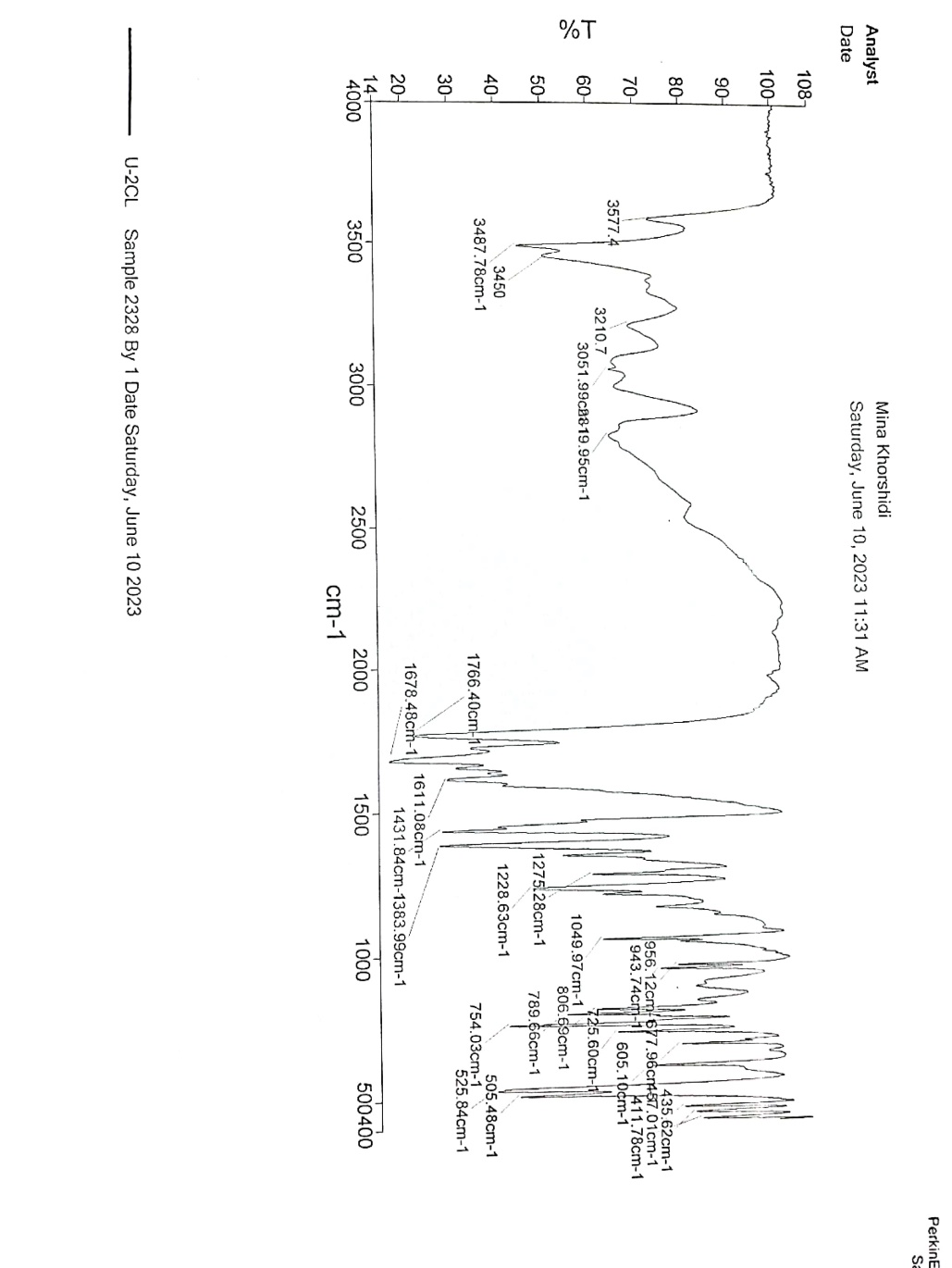


FT-IR spectrum of **2h**


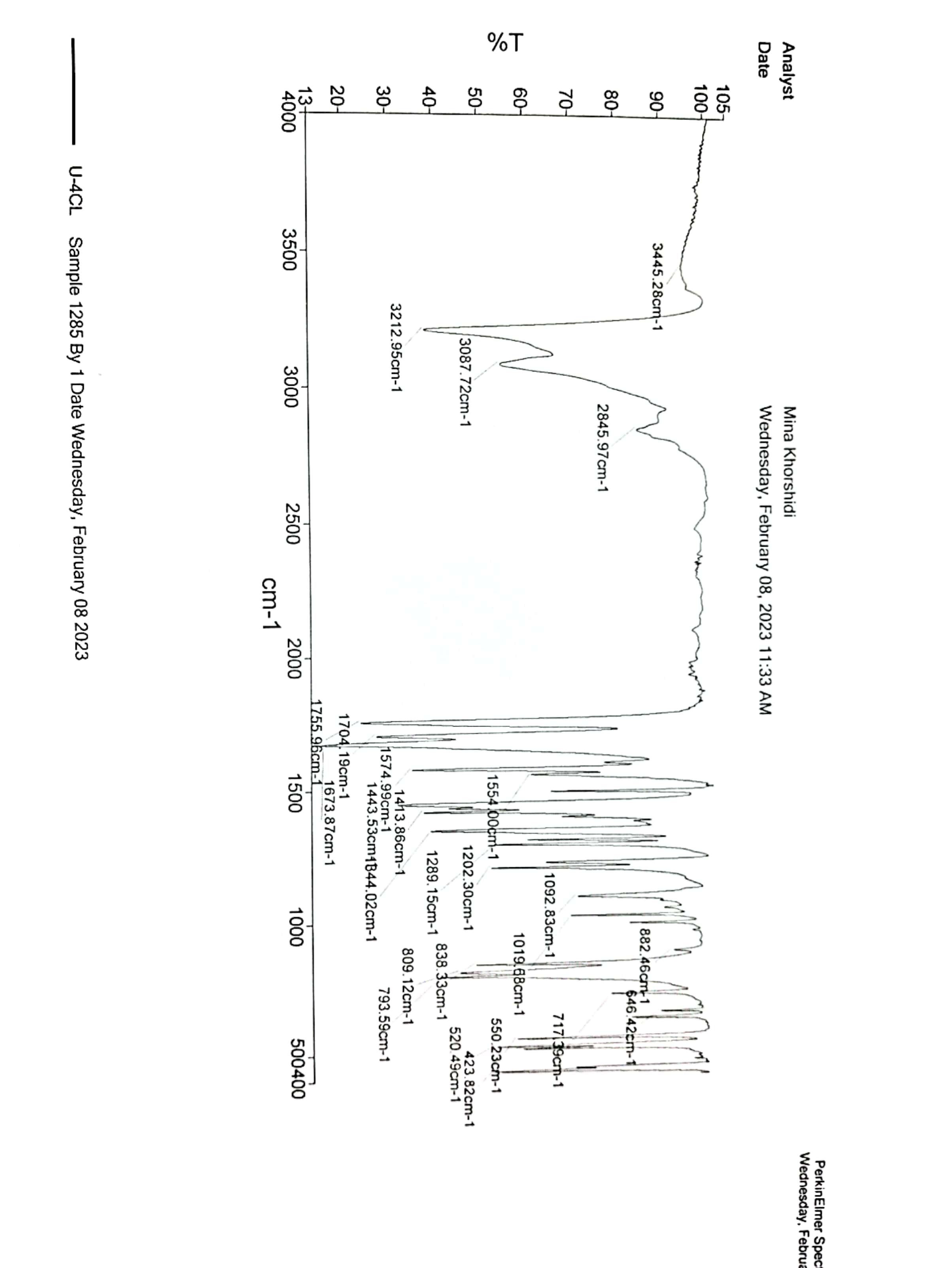


FT-IR spectrum of **2i**


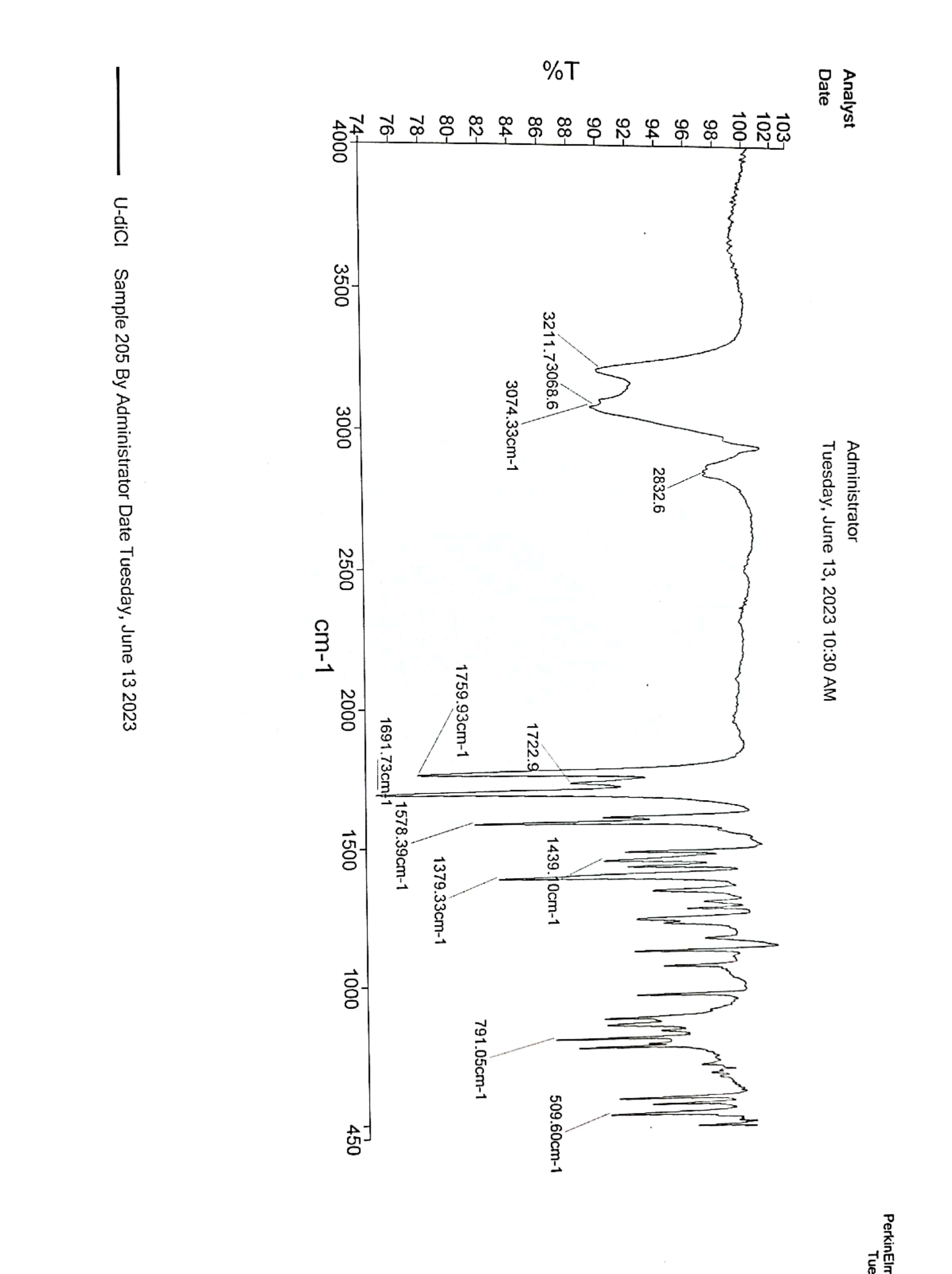


FT-IR spectrum of **2j**


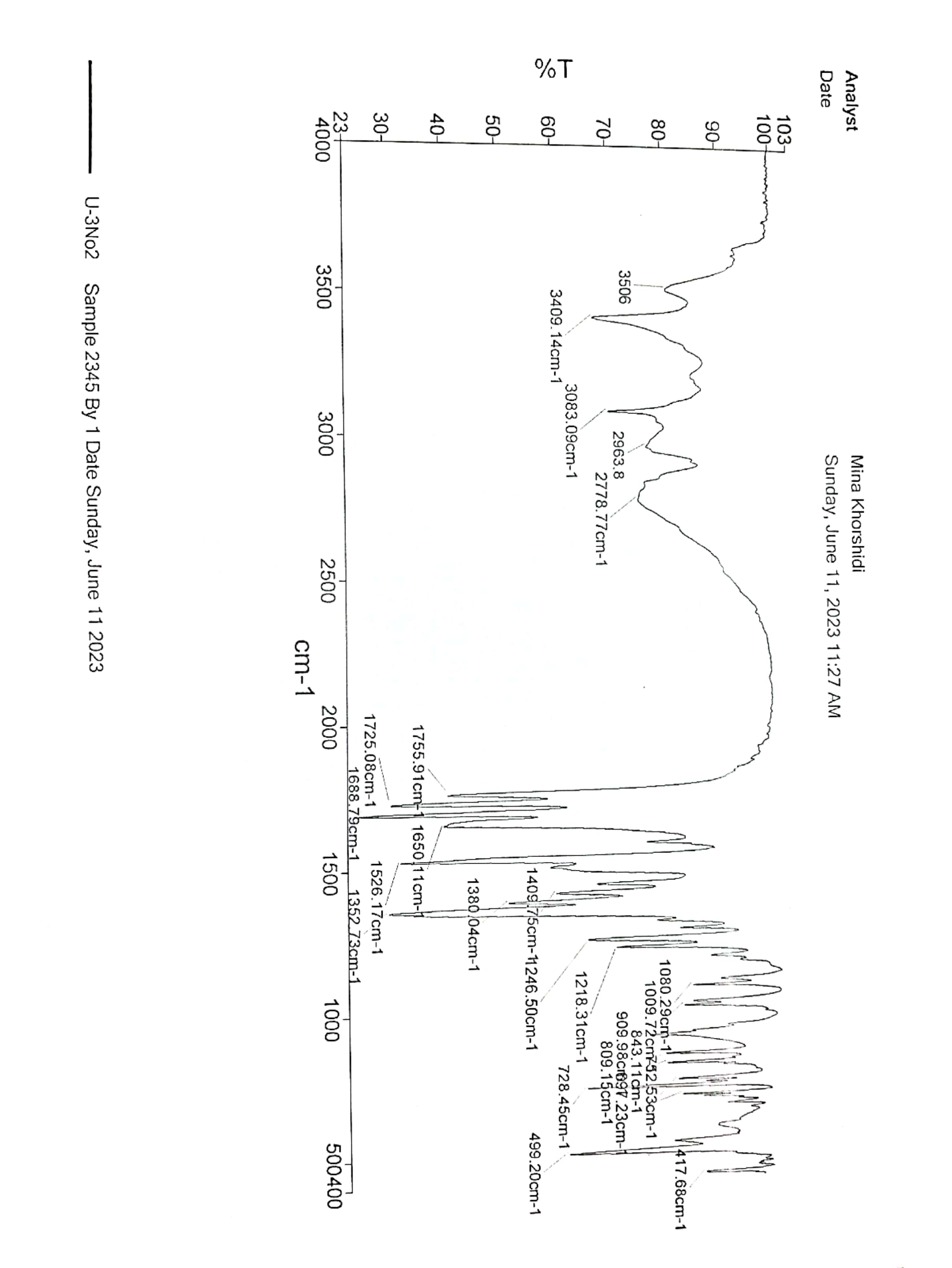


FT-IR spectrum of **2k**


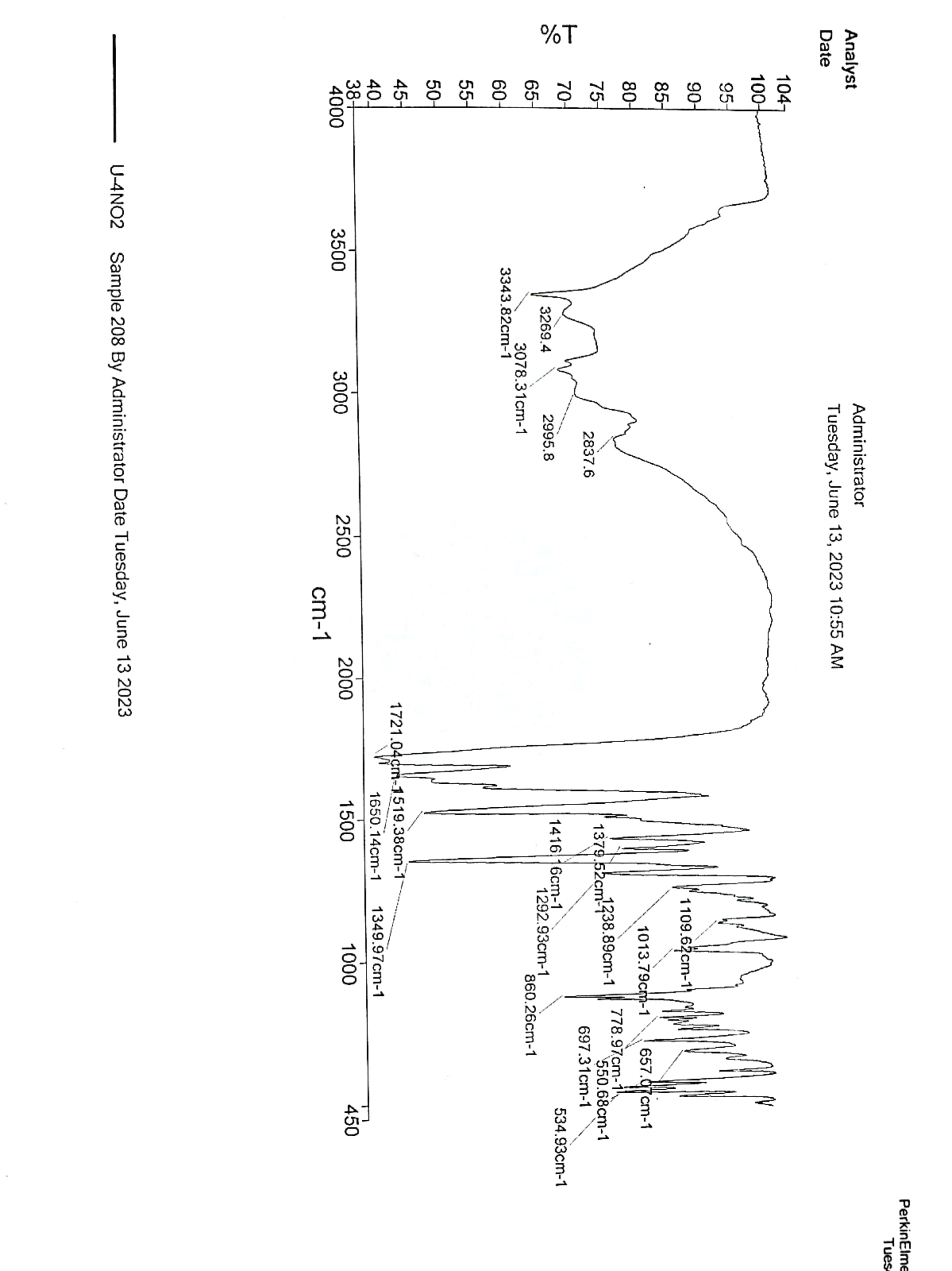


FT-IR spectrum of **2l**


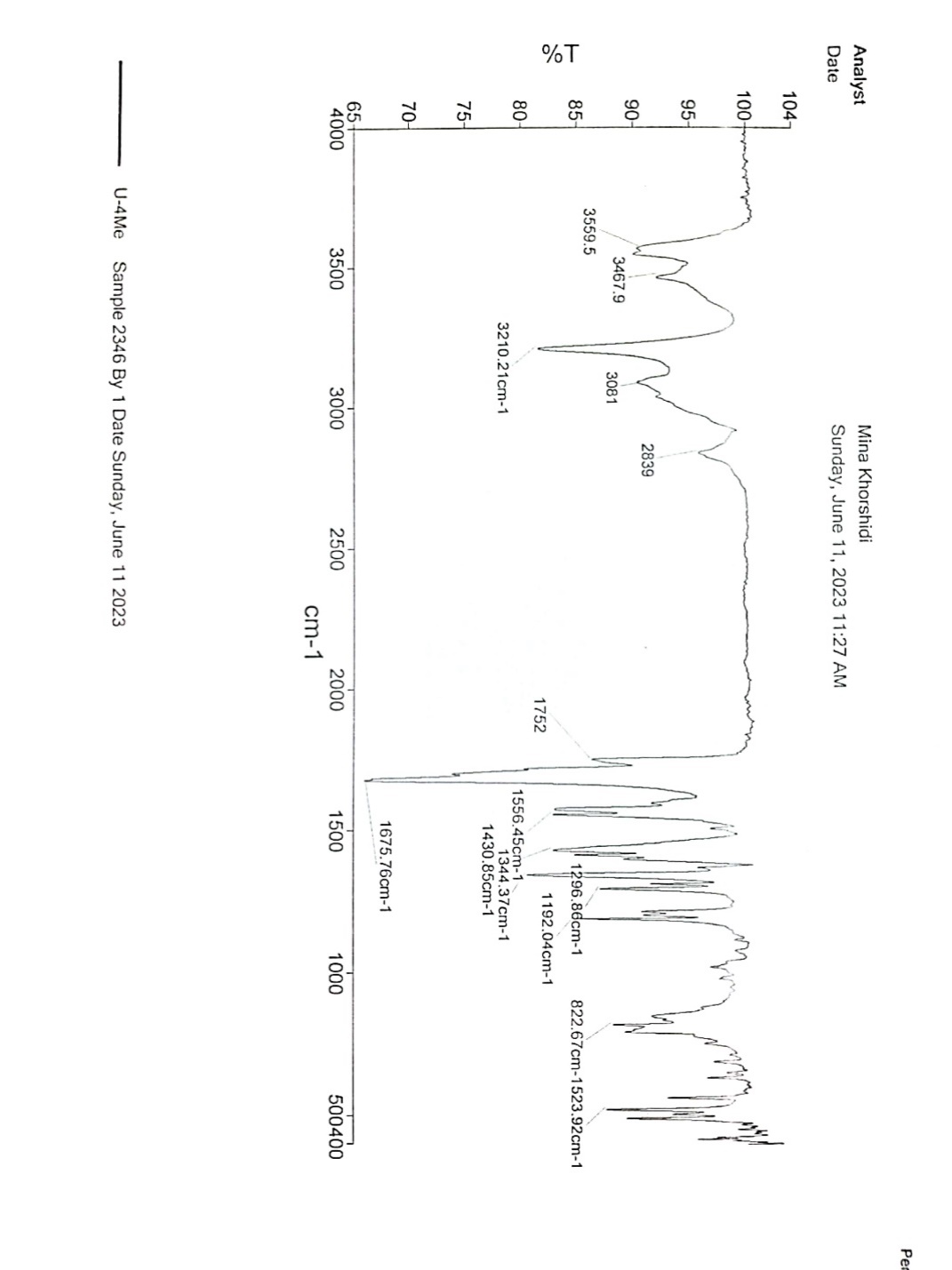


FT-IR spectrum of **2m**


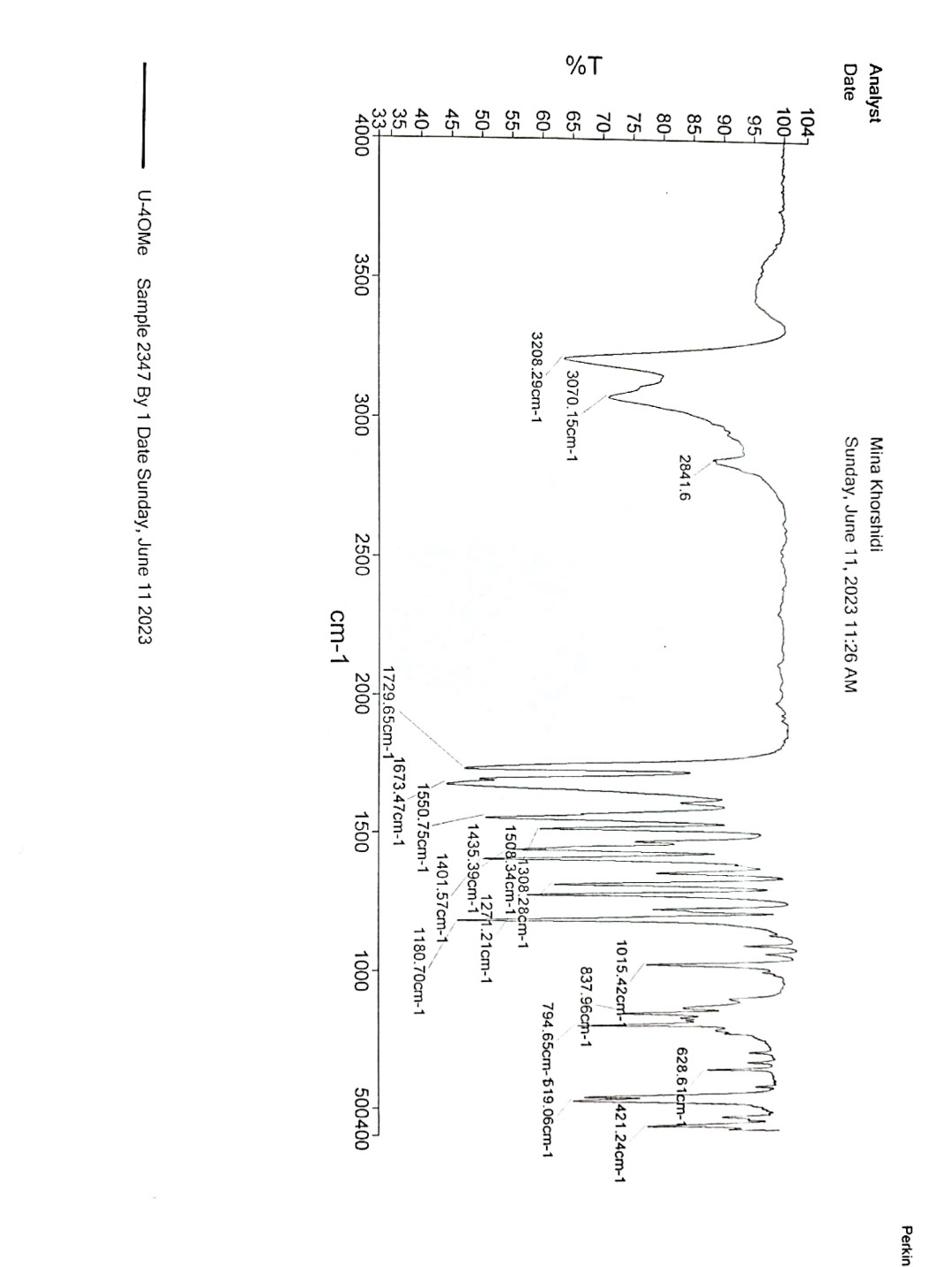


FT-IR spectrum of **2n**


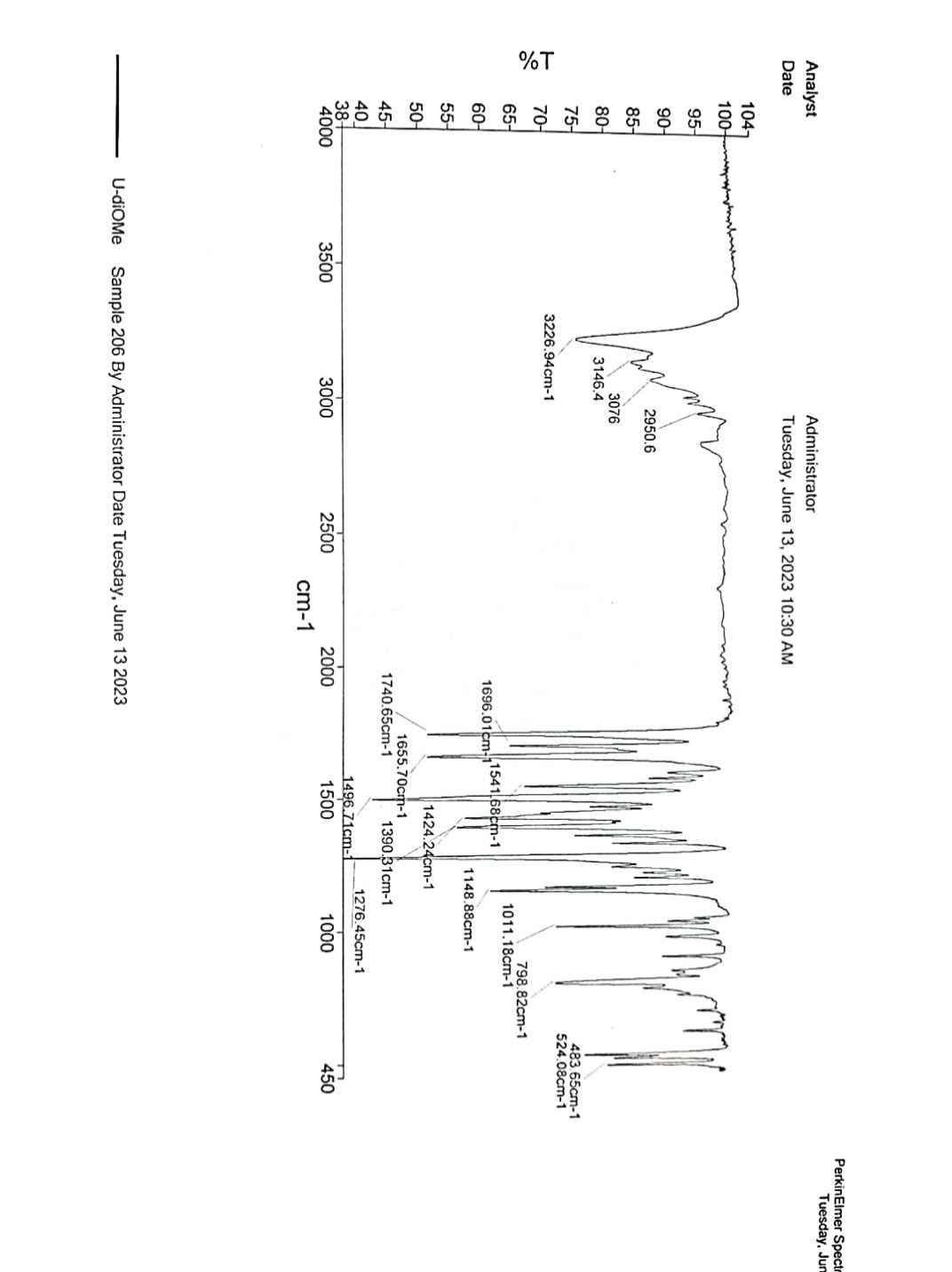


FT-IR spectrum of **2o**


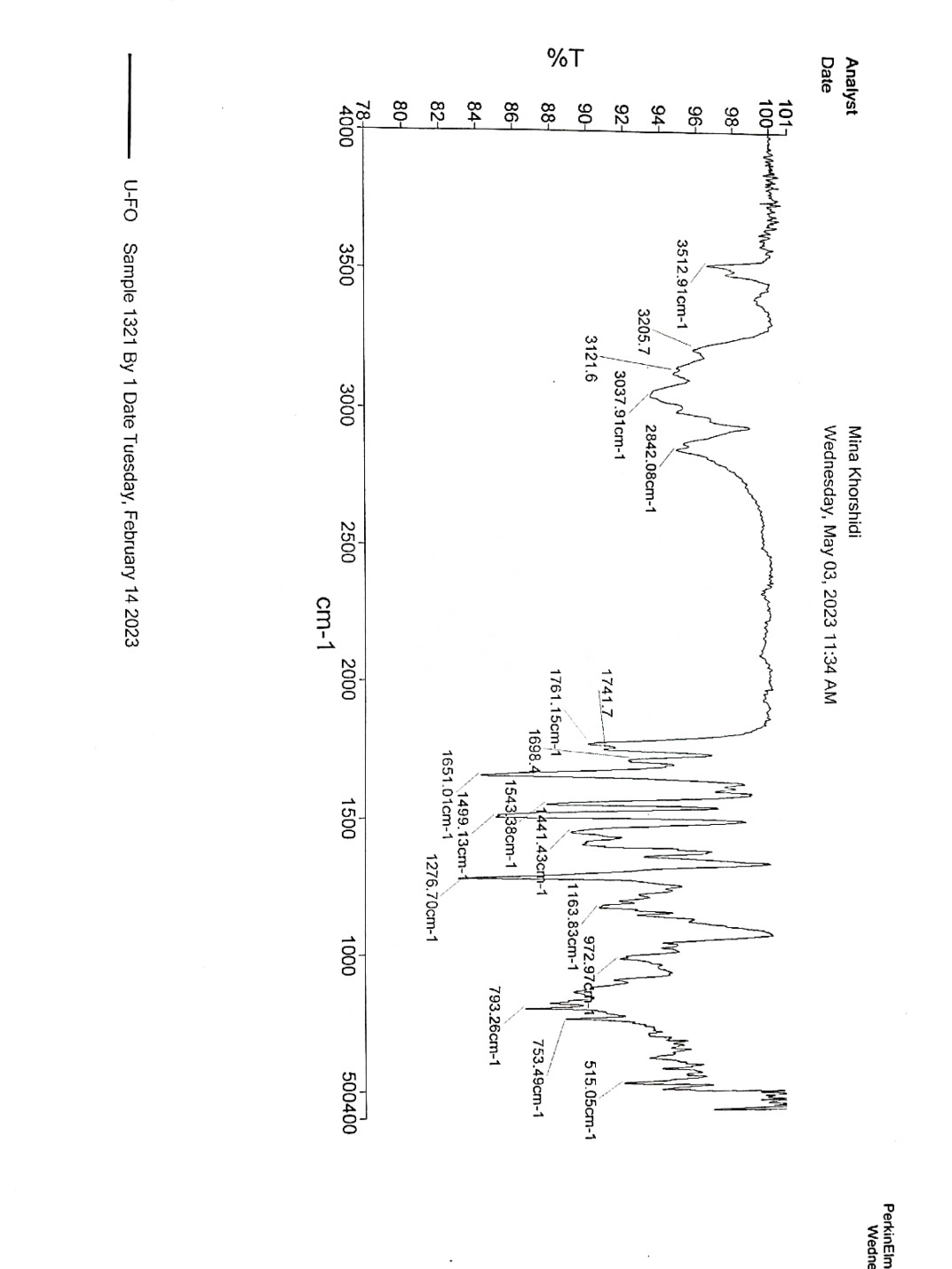


FT-IR spectrum of **2p**


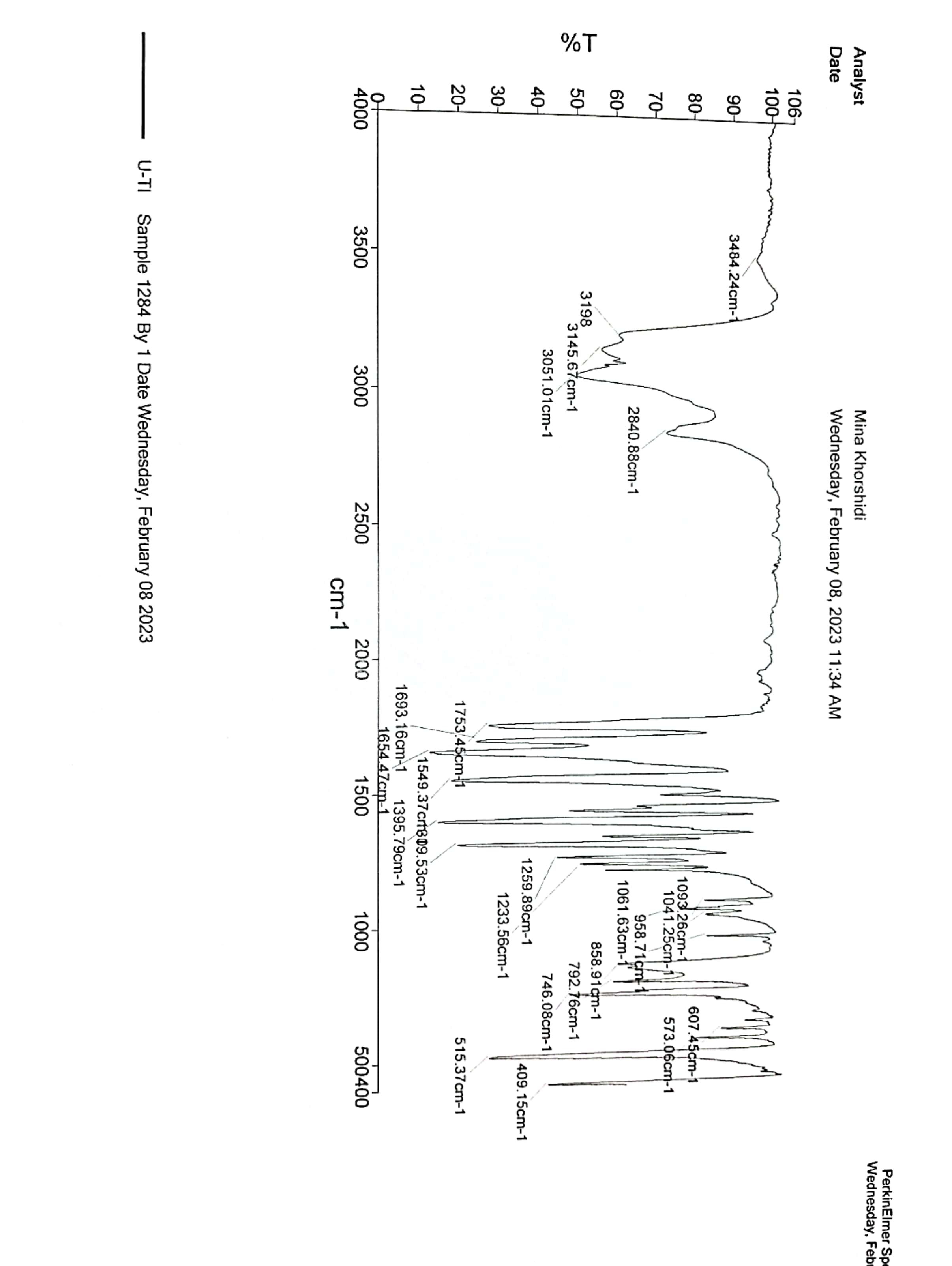


FT-IR spectrum of **3a**


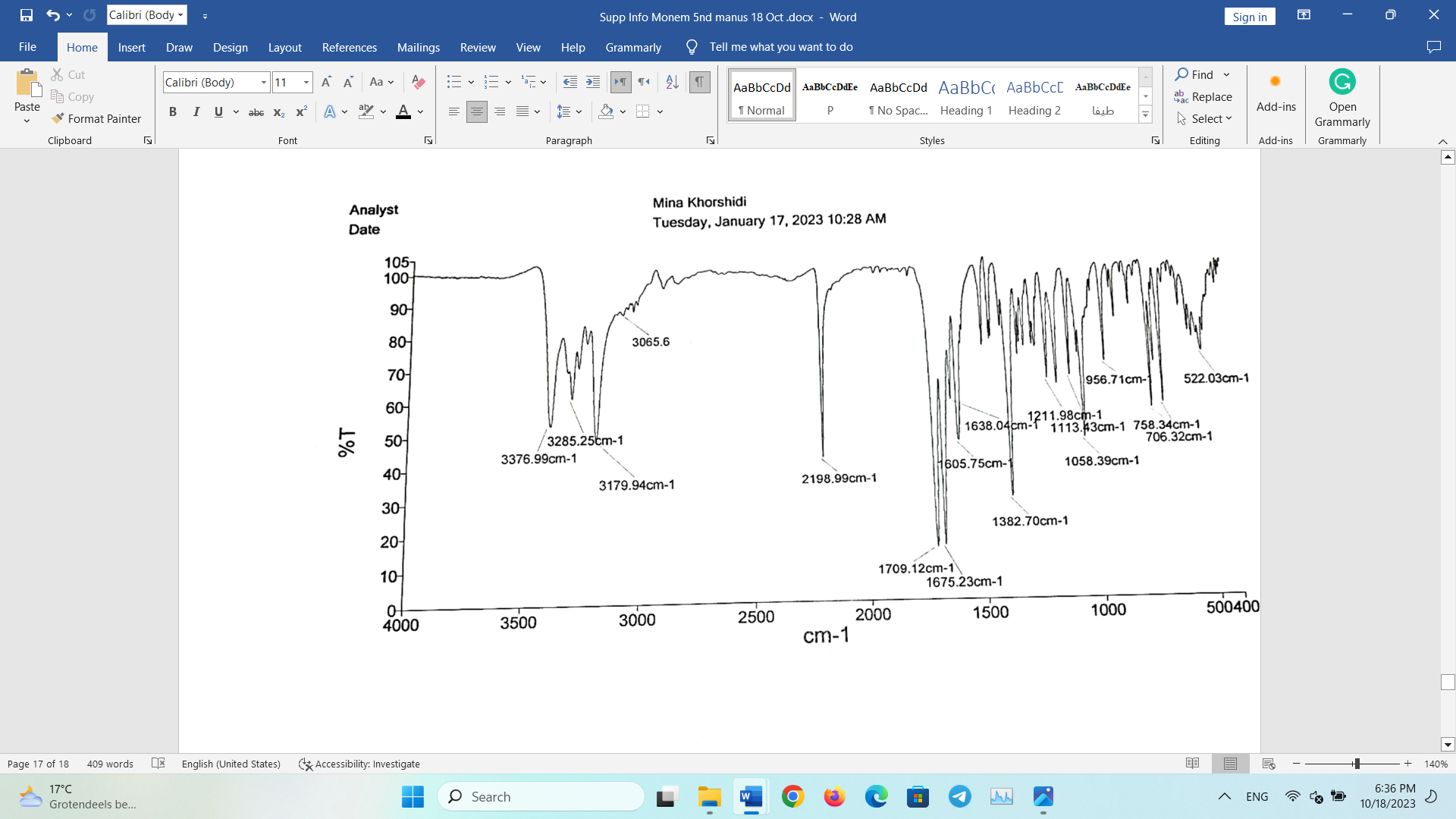


FT-IR spectrum of **3b**


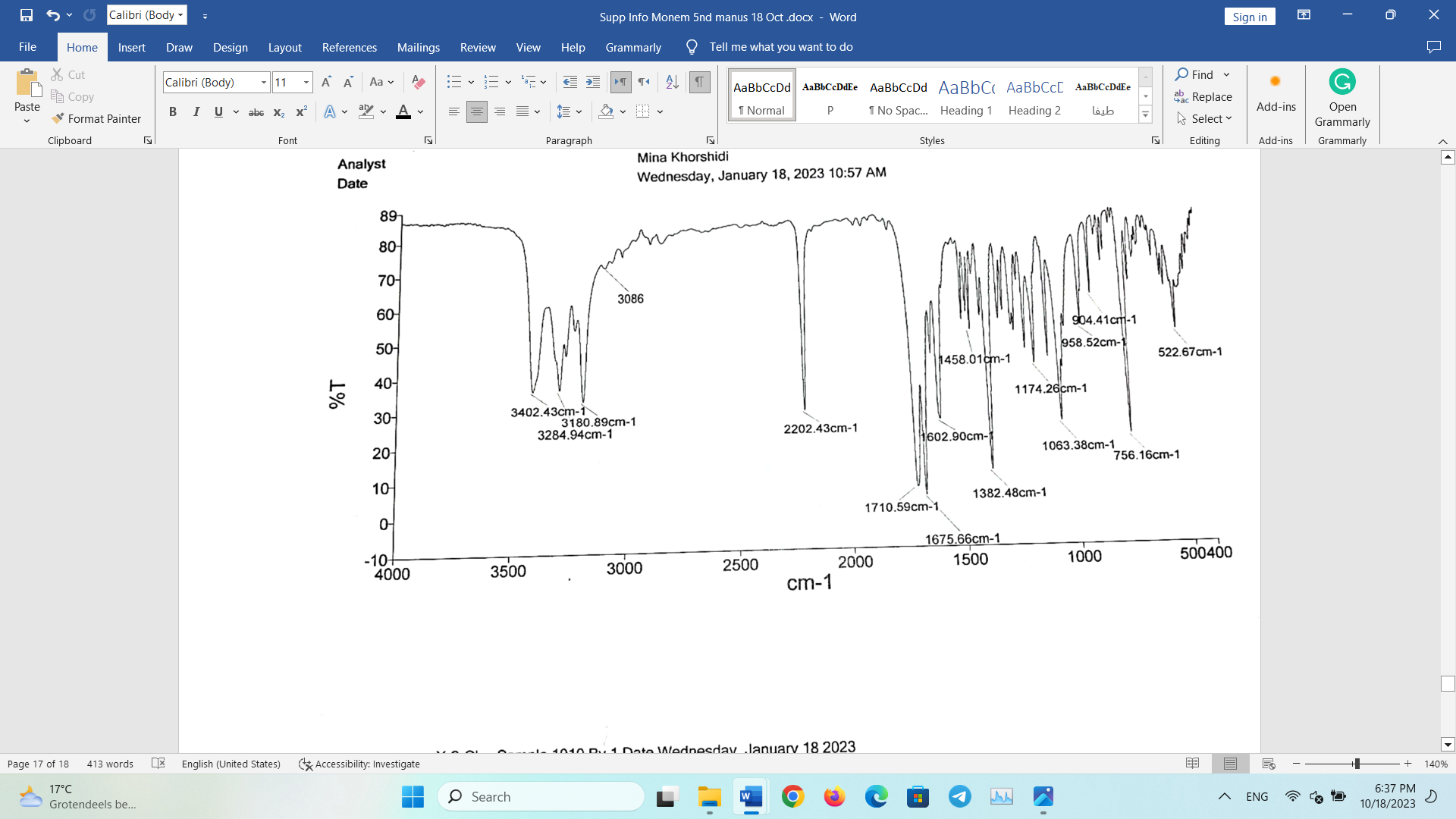


^1^H NMR spectrum of **3b**

FT-IR spectrum of **3c**


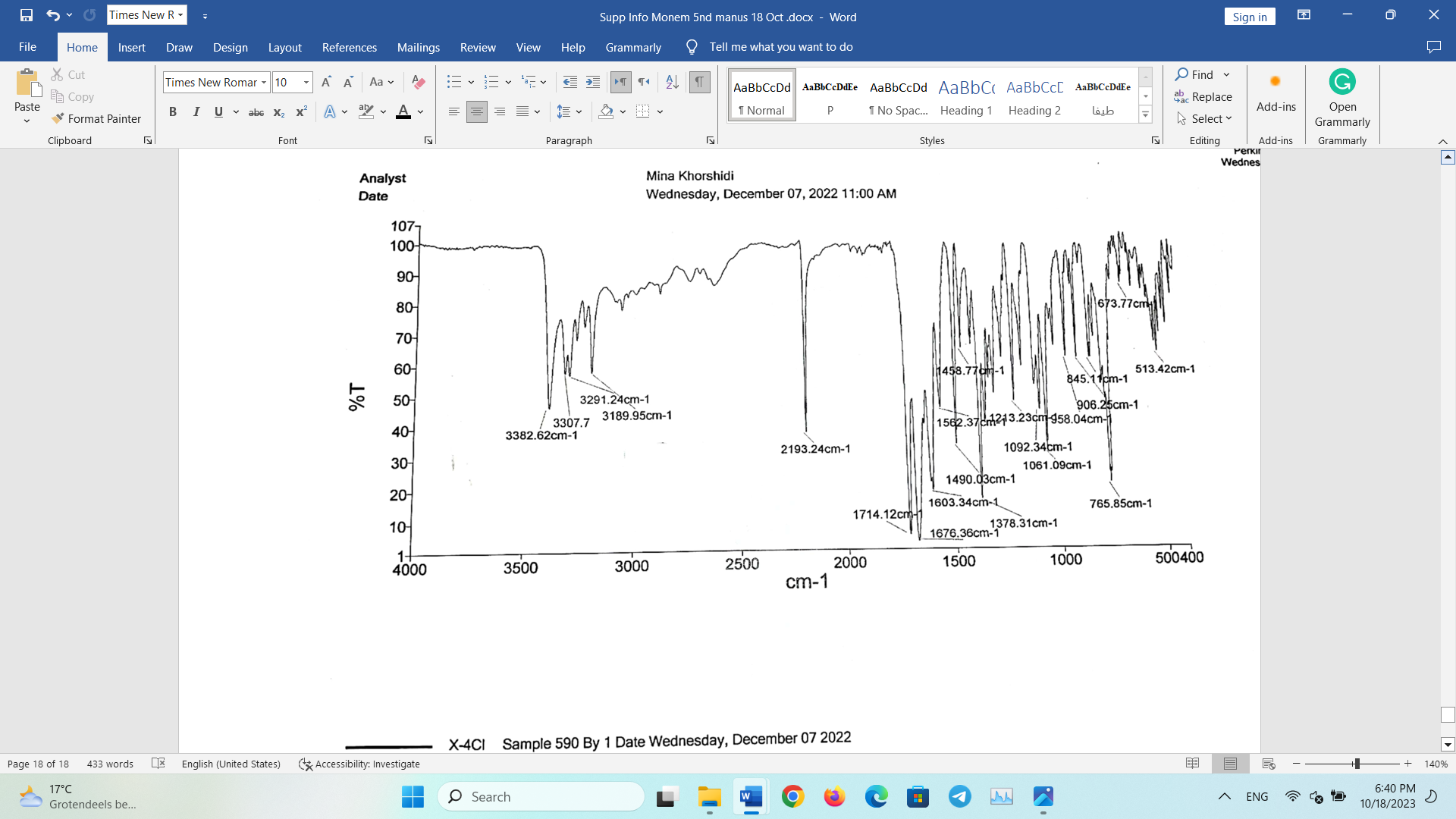


FT-IR spectrum of **3f**


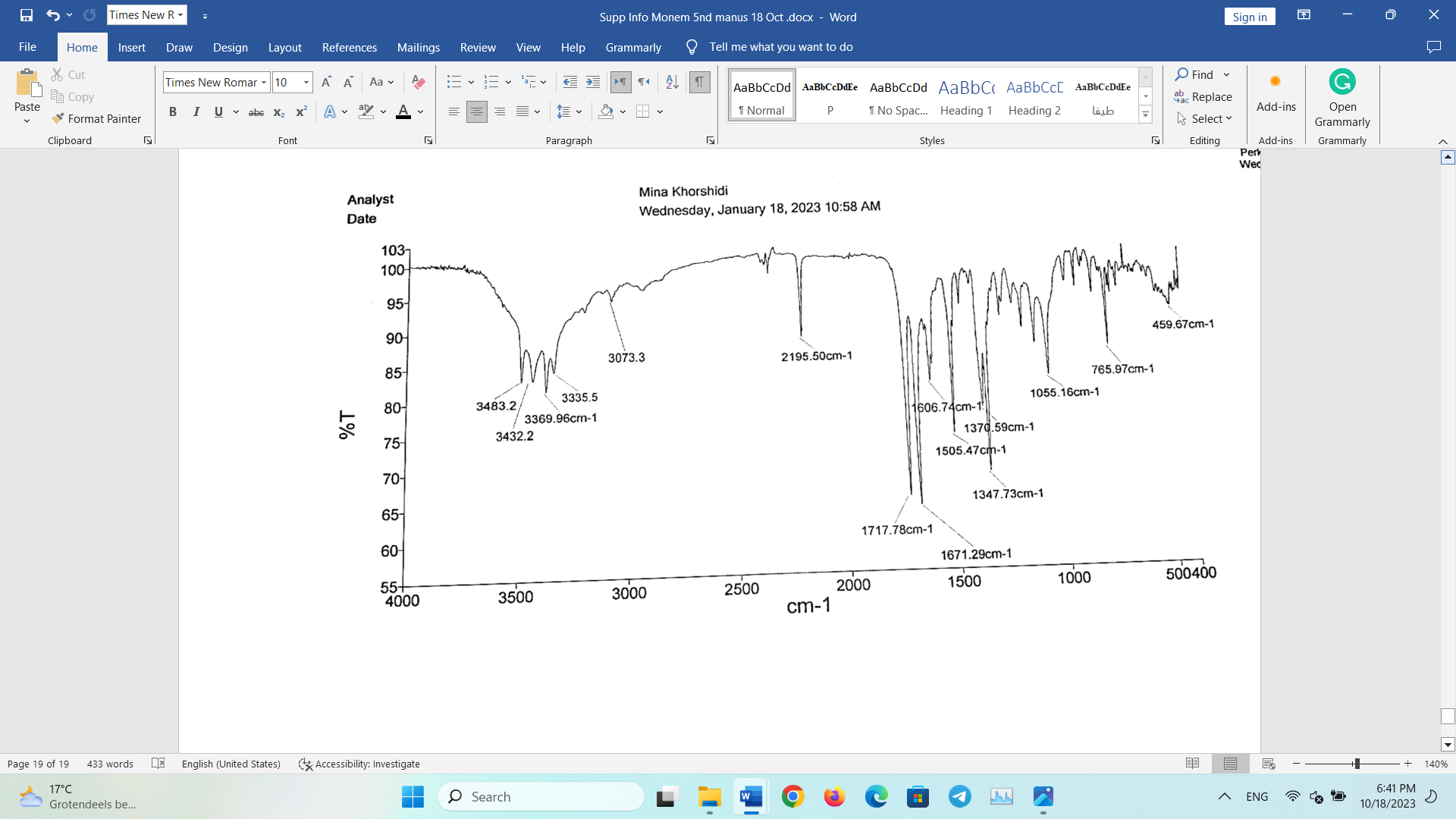


FT-IR spectrum of **3g**


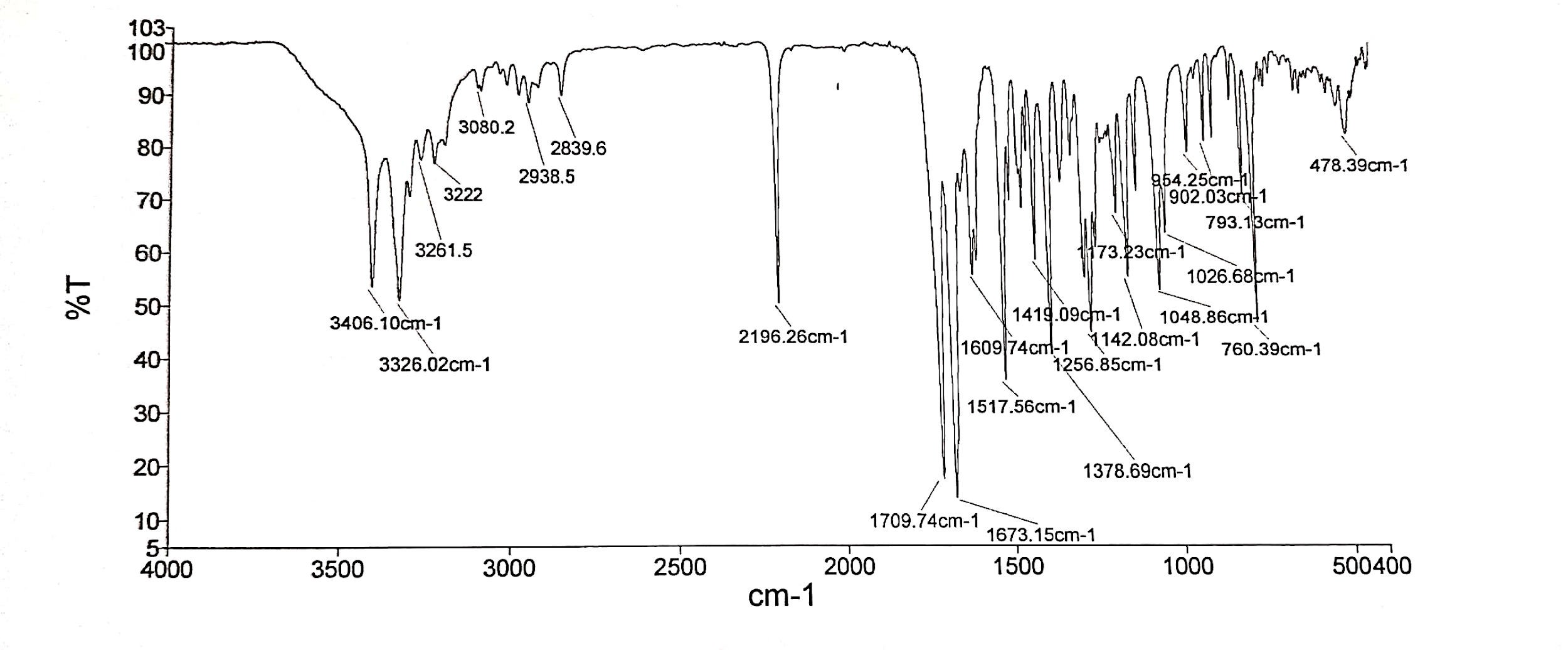


^1^ HNMR spectrum of **3g**


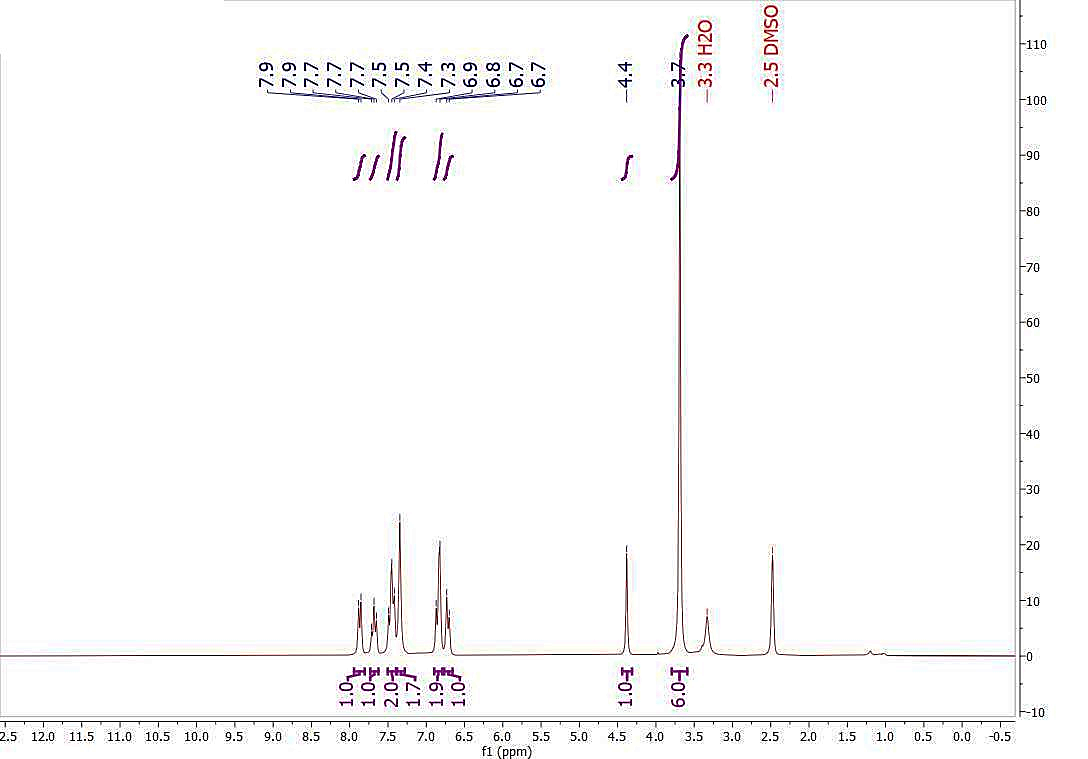


^13^ CNMR spectrum of **3g**


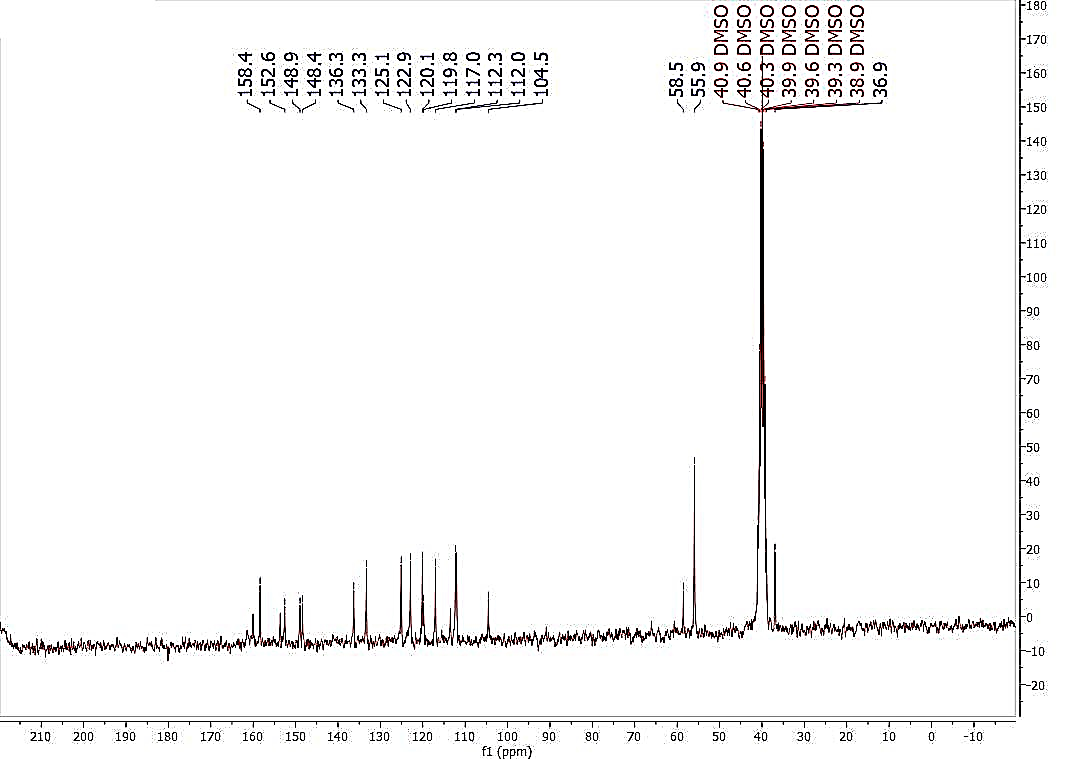


FT-IR spectrum of **3h**


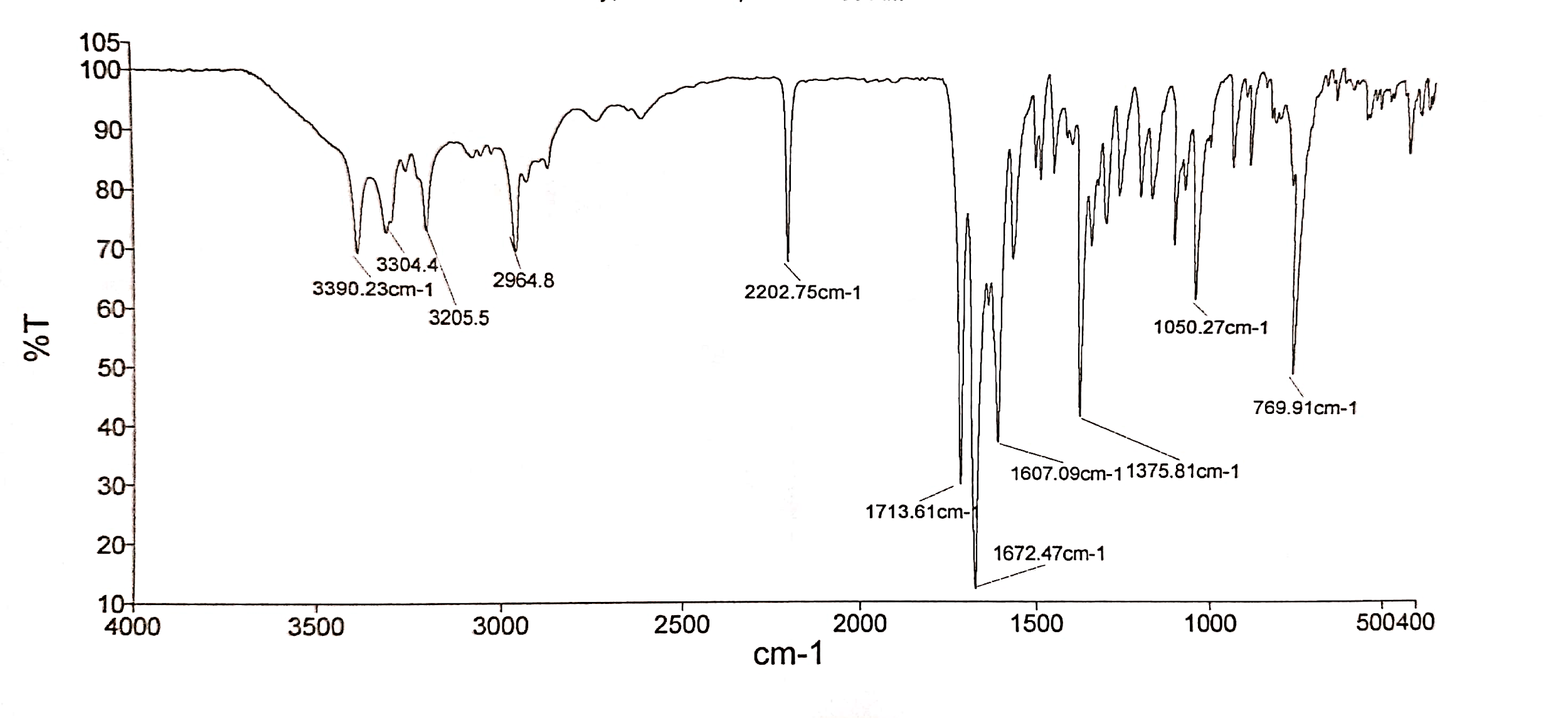


FT-IR spectrum of **3i**


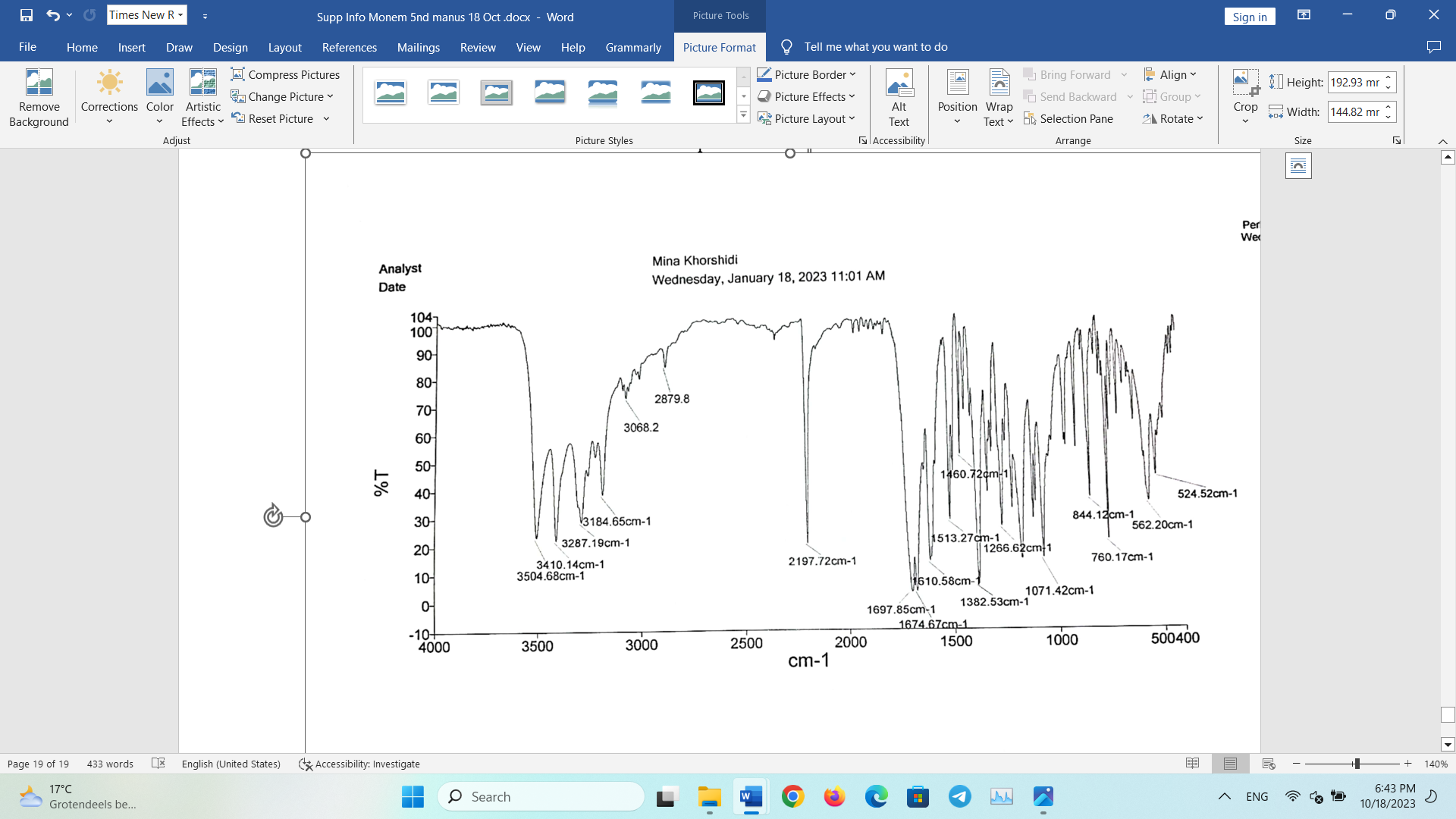


FT-IR spectrum of **3j**


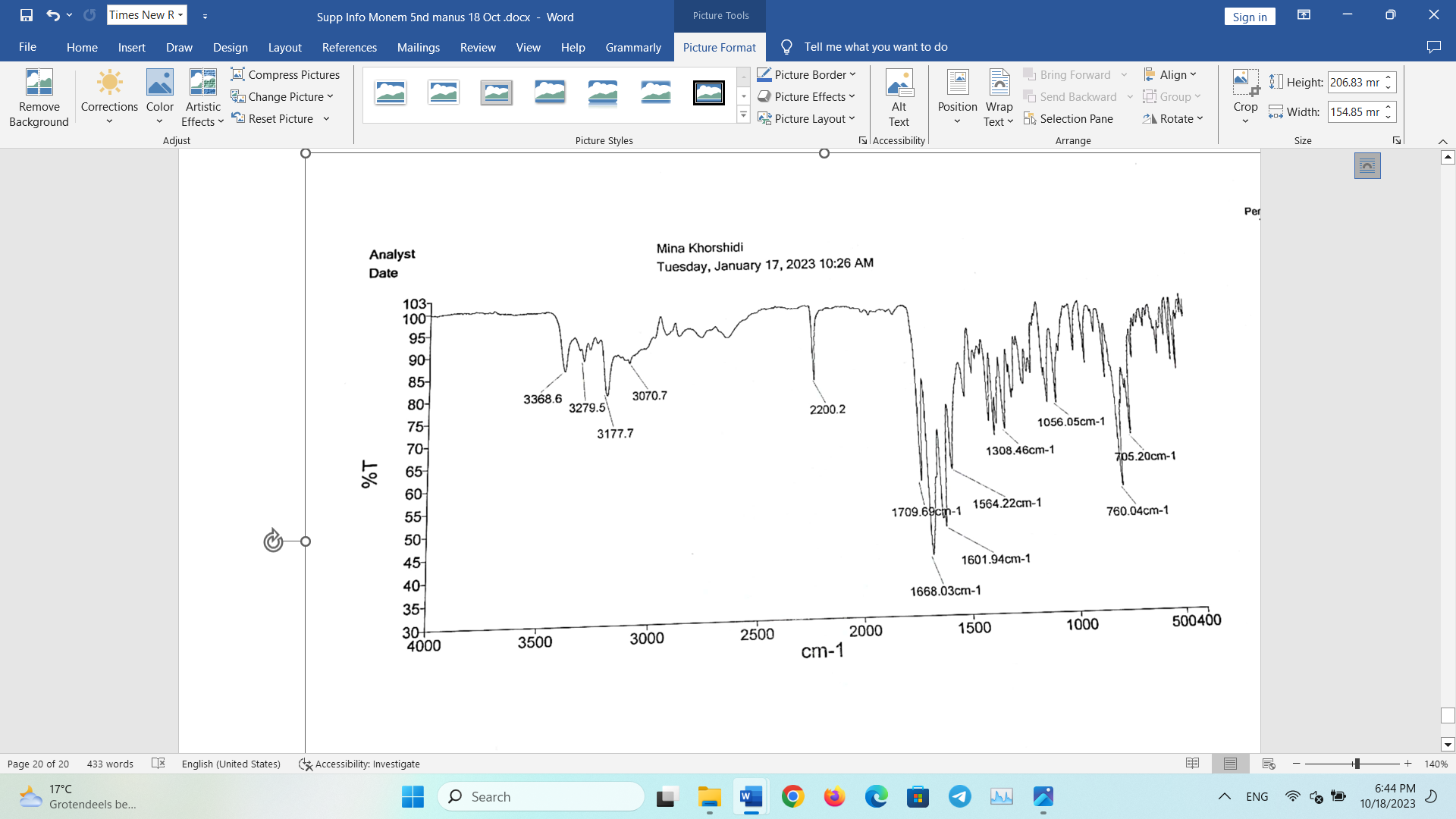

Supplement: Supplementary file 1 — Supplementary Material 1. [file 13065_2024_1227_MOESM1_ESM.docx]
